# Synthesis and Characterization of Functionalized Silica Particles: A Course-Based Undergraduate Research Experience in Materials Chemistry

**DOI:** 10.1021/acsomega.5c05617

**Published:** 2025-10-03

**Authors:** Marco Bell, Elizabeth K. Dierlam, Cayden Smith, Luke A. Wolf, Abby R. Jennings

**Affiliations:** † Department of Chemistry, 2611 United States Air Force Academy, 2355 Fairchild Drive, USAF Academy, Colorado 80840, United States; ‡ Heersink School of Medicine, University of Alabama at Birmingham, 1670 University Blvd, Birmingham, Alabama 35294, United States

## Abstract

A course-based undergraduate research experience demonstrating the synthesis and functionalization of silica particles prepared using a modified Stöber method was implemented. Silica particles were functionalized utilizing co-condensation and delayed condensation procedures. FT-IR and thermogravimetric analyses showed that the functionalization methods were successful. Dynamic light scattering and scanning electron microscopy indicated that both functionalization methods produced nanometer sized particles that aggregated in solution. Glass slides were spin coated with suspensions of unfunctionalized nanometer sized silica particles and particles functionalized by both methods. Optical profilometry and atomic force microscopy indicated that all samples had less macroscale surface roughness than nanoscale surface roughness and that the functionalized particles had over two times more nanoscale surface roughness than the unfunctionalized particles. Water contact angle analysis indicated that the glass slides coated with the functionalized particles were less hydrophilic than the glass slides coated with the bare particles.

## Graphical abstract

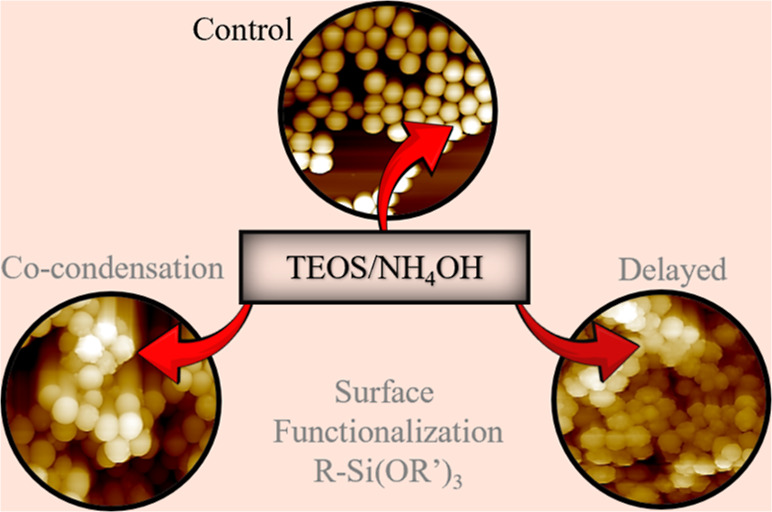

## Introduction

Nanotechnology is an important area of study in science and engineering fields. Nanotechnology deals with preparing functional materials on the nanometer scale (1–100 nm) utilizing top-down methods, such as ball-milling or bottom-up methods, including solution–gelation (sol–gel) chemistries. Nanomaterials are unique in that they have a high-surface area to volume ratio, yielding materials with distinctive properties when compared to bulk materials of the same chemical composition.^1^

One class of nanomaterial that has received continued interest in the scientific community is silicon based. These include but are not limited to aerogels, polyhedral silsesquioxanes (POSS), polymers, and silica nanoparticles; encompassing both amorphous and mesoporous particles (MSNs). These materials can easily be prepared using sol–gel methods, have tailorable mechanical and chemical properties, and show excellent biocompatibility.^2^ With their size regime, low-toxicity, and ease of chemical modification, silica nanoparticles are often utilized for advanced applications, such as drug delivery, and there are a number of recent review articles highlighting this.^3–5^ Amorphous silica nanoparticles can easily be prepared by the hydrolysis and condensation of tetraalkoxysilanes (Si­(OR)_4_), like tetraethylorthosilcate (TEOS) and tetramethylorthosilicate (TMOS), often referred to as the Stöber method.^6^ Furthermore, the size of the nanoparticles can be tailored through simple modifications in reaction conditions or concentrations. This produces SiO_2_ nanoparticles with hydroxyl groups at their surface. These surface silanols serve as functionalization sites, where reactive silanes, such as alkoxysilanes or chlorosilanes, can be attached, yielding functionalized silica nanoparticles.

Although reported on heavily in the scientific literature, the use of amorphous silica nanoparticles in an academic teaching setting is much less utilized, especially given their ease of synthesis and chemical modification.^7^ As a result, a course-based undergraduate research experience (CURE) was designed to expose undergraduates to the important field of nanomaterials. As highlighted by a recent review, implementation of CUREs in undergraduate chemistry curriculum has gained significant traction over the past decade.^8^ CUREs are mutually beneficial for students and faculty alike. Faculty benefit from integrating research interest with teaching efforts, which can enhance scholarly productivity, support undergraduate researcher recruitment, generate positive artifacts for promotion, and many others.^9^ For students, many studies indicate that CUREs enhance scientific proficiency and communication, improve retention in STEM fields, and eliminate time constraints typically seen within the research setting, along with other benefits.^10,11^ Although some discipline-specific components of CUREs exist, the consensus is that they contain 5 common elements: discovery of new knowledge, student collaboration, iteration, broad relevance, and engagement in scientific practices.^12,13^

The CURE developed emphasizes the synthesis, functionalization, and characterization of silica nanoparticles by utilizing sol–gel methods and a modified Stöber method. After providing a handout with basic procedural details (see the Supporting Information), students had the freedom to select which trialkoxysilane (R–Si­(OR’)_3_) their particles were functionalized with ((3-aminopropyl)-trimethoxysilane, *n*-octryltrimethoxysilane, (tridecafluoro-1, 1, 2, 2-tetrahydrooctyl) triethoxysilane, or 3-(acryloxypropyl) trimethoxysilane). Next, the optimal amount of functionalized trichlorosilane (up to 500 μL) was investigated. The goal was for the students to identify what parameters were sufficient to alter the measured properties compared to a control but not enough to induce gelation or aggregation. Students also utilized the chemical literature to identify and develop spin-coating parameters for surface analysis. The impacts on variations in these modifications on the particles and their spin-cast films were then investigated through a variety of common material characterization methods.

The main learning outcomes of the CURE were for students to build confidence in advanced material characterization methods, including independent operation, data analysis, and data interpretation, use the chemical literature to develop/modify experimental procedures, and improve scientific writing and communication. These were assessed utilizing a midcycle progress review, literature article submission, and a final written lab report (see the Supporting Information). The CURE that resulted in the most complete and consistent results was obtained from using 100 μL of *n*-octryltrimethoxysilane via co-condensation and delayed condensation. Those results are presented here-in.

## Materials and Methods

### General

All reagents were purchased from commercial sources and used as received, unless stated otherwise. The following chemicals were used in this laboratory: 28–30% ammonium hydroxide solution, absolute ethanol, tetraethyl orthosilicate (TEOS), and *n*-octyltrimethoxysilane (*n*-OTMS). A 9 M ammonium hydroxide solution was prepared and used as the catalyst solution.

### Instrumentation

Particle size analysis was performed by dynamic light scattering (DLS), using a Malvern Zetasizer Nano ZS instrument with 12 mm disposable cuvettes (DTS0012). Silicon dioxide particles or colloids were used as the material, and ethanol (viscosity = 1.1440 cP, refractive index = 1.36) was selected as the dispersant. Samples were equilibrated for 120 s, and measurements were collected in triplicate with a backscatter of angle of 173° at 20 °C. Scanning electron microscopy (SEM) was performed on a Tescan Vega 3. Liquid samples were carefully placed on standard SEM stubs with the double-sided carbon tape. Once dry, the samples were sputter coated with a 5 nm gold coating by using a Quorum Technologies X150R sputter coater. SEM images were acquired with an accelerating voltage of 30 kV and a magnification of 5 kx. Fourier transform infrared (FT-IR) spectra were recorded on a Thermo Scientific Nicolet iS20 instrument equipped with a Smart iTX ATR accessory. Spectra were collected over 16 scans at a resolution of 4 cm^–1^ and a range of 3800–500 cm^–1^. Thermogravimetric analysis (TGA) was collected on a TA 5500, utilizing platinum pans. Data were collected under nitrogen, from ambient up to 900 °C at a ramp rate of 10 °C/min. Data analysis was performed by using TRIOS software. A VTC-100 vacuum spin coater was used to make films of the particle suspensions. The spin coating process was achieved in two steps; first at 500 rpm for 10 s, followed by 2000 rpm for 60 s. Surface roughness of the spin-coated glass slides was measured at five randomly selected 800 × 800 μm^2^ areas using a Bruker GT noncontact optical surface profilometer. The average roughness (Sa) of the surfaces was measured. Average surface roughness (Ra) of the spin-coated glass slides and morphology of the nanoparticles were obtained by atomic force microscopy (AFM) using a Park Systems NX10 AFM. An aluminum-coated silicon cantilever with a force constant of 42 N/m, a resonance frequency of 330 kHz, and a radius less than 10 nm was used to measure height topographies. Measurements were collected at 512 × 512 or 1024 × 1024 pixels in the noncontact mode with an area measuring 10 × 10 μm^2^. Data analysis of the height images was performed using XEI software, version 5.1.6. Surface roughness of the spin-coated glass slides was measured at five randomly selected 2.5 × 2.5 μm^2^ areas. Water contact angle (WCA) analysis of the spin-coated glass slides was performed on a MSE PRO standard contact angle meter/goniometer. WCA measurements were taken at five randomly selected areas on the coated slides.

### Synthesis and Functionalization of Silica Particles

#### Control

A 25 mL scintillation vial, equipped with a magnetic stir bar, was charged with 10 mL of the catalyst solution, 5 mL of absolute ethanol, and 0.5 mL of TEOS. The solution was vigorously stirred for 16 h, under ambient conditions. The particles were isolated via centrifugation and dried (136 mg recovered).

#### In Situ Functionalization 1 (IF-1)

A 25 mL scintillation vial, equipped with a magnetic stir bar, was charged with 10 mL of the catalyst solution, 5 mL of absolute ethanol, and 0.5 mL of TEOS. After stirring under ambient conditions for about 5 min, 0.1 mL of *n*-OTMS was added. The solution was vigorously stirred, under ambient conditions, for 16 h. The particles were isolated via centrifugation and dried (199 mg recovered).

#### In Situ Functionalization 2 (IF-2)

A 25 mL scintillation vial, equipped with a magnetic stir bar, was charged with 10 mL of the catalyst solution, 5 mL of absolute ethanol, and 0.5 mL of TEOS. The solution was vigorously stirred, under ambient conditions, for 16 h. Then, 0.1 mL of *n*-OTMS was added to the scintillation vial and vigorously stirred for an additional 16 h under ambient conditions. The particles were isolated via centrifugation and dried (207 mg recovered).

Table 1 summarizes the reagents, amounts, reaction times, and conditions for the control, IF-1, and IF-2.

**Table 1:** Experimental Summary for the Synthesis of the Control, IF-1, and IF-2^d^

**sample**	**catalyst (mL)**	**ethanol (mL)**	**TEOS (mL)**	*n* **-OTMS** **(μL)**	**functionalization delay (h)** ^a^	**total time (h)** ^b^
control	10	5	0.5	0	^c^	16
IF-1	10	5	0.5	0.1	0.08	16
IF-2	10	5	0.5	0.1	16	32

a The functionalization delay is the period of time between the start of the condensation reaction of TEOS and the addition of *n*-OTMS.

b Total time is the full duration of TEOS condensation.

c The control sample was not functionalized with *n*-OTMS.

d All samples were prepared under ambient conditions.

### Washing and Drying Protocol

The particle suspensions were transferred to preweighed centrifuge tubes and centrifuged at 2500 rpm for 10 min. The supernatants were discarded, and the pellets were resuspended in 8.0 mL of absolute ethanol. The centrifugation and resuspending of the particles were repeated two more times. The final pellets were placed in a vacuum oven at 85 °C for 48 h. The dry particles were characterized by FT-IR and TGA.

### Spin Coating

Particle suspensions (10 mg/mL) in absolute ethanol were used for spin coating glass slides measuring 2.50 × 2.50 cm^2^. Initially, particle analysis was performed on the resuspended samples using DLS and SEM. The suspension (300 mL) was placed on the glass slide, and then a two-stage spin coating was performed; first at 500 rpm for 10 s, followed by 2000 rpm for 60 s. Samples were dried under ambient conditions before surface analysis. Surface analysis was performed by optical profilometry, AFM, and WCA.

## Results and Discussion

Three separate sets of reaction conditions were utilized in preparing nanometer-sized silica particles utilizing a modified Stöber method.^6,14^ Initially a control sample was prepared using tetraethyl orthosilicate, or TEOS, as the only alkoxysilane undergoing hydrolysis and condensation. In situ functionalization with *n*-octyltrimethoxysilane (*n*-OTMS) was achieved in two different one-pot reactions; in the first (IF-1), TEOS and *n*-OTMS were added at the same time such that the *n*-octyl functional group would be directly incorporated into the growing silica network.^15^ In the second one-pot reaction, TEOS was initially added, and the silica particles were allowed to form. After 16 h, the *n*-OTMS was added for surface functionalization, Scheme 1.

**Scheme 1:**
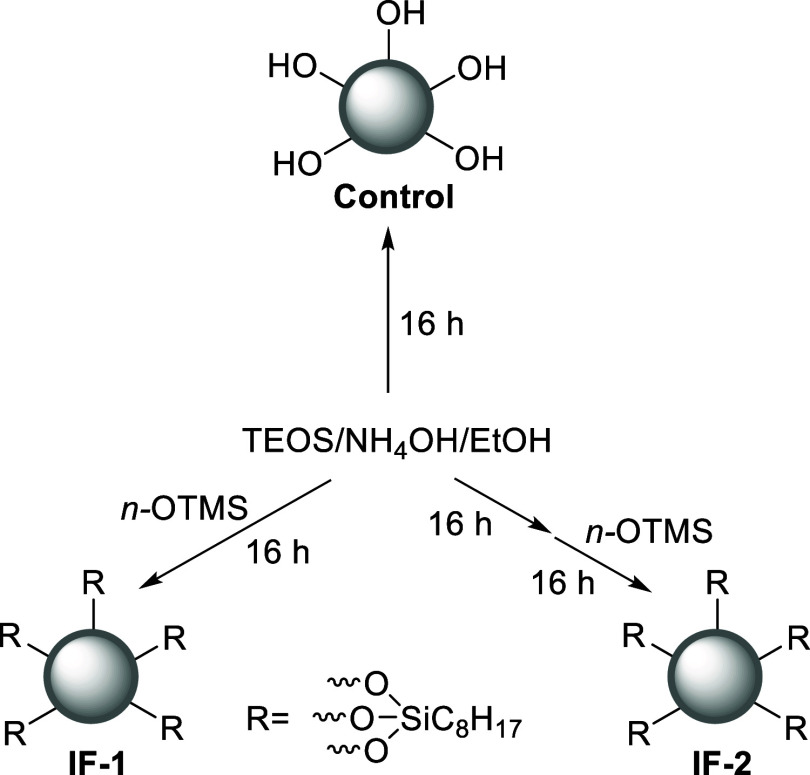
Synthetic Method Used to Obtain the Control, IF-1, and IF-2.

In all cases, suspensions were obtained and particles were isolated and purified following a centrifugation and washing protocol.^16^ Oven-dried particles, particles suspended in absolute ethanol, and glass slides spin coated with particle suspensions were analyzed by a variety of characterization methods.

Thermal gravimetric analysis (TGA) was performed on vacuum-dried particle samples in order to investigate their organic content and the degree of functionalization, Figure 1.^17^ All samples showed a small mass loss from ambient up to 200 °C, with the majority coming off below 100 °C. This is likely due to a small amount of physically absorbed water.^18,19^ This mass loss was less than 5% for all samples, and both IF-1 and IF-2 absorbed less water than the control. This could be attributed to the presence of more hydrophobic *n*-octyl functional groups.

**Figure 1:**
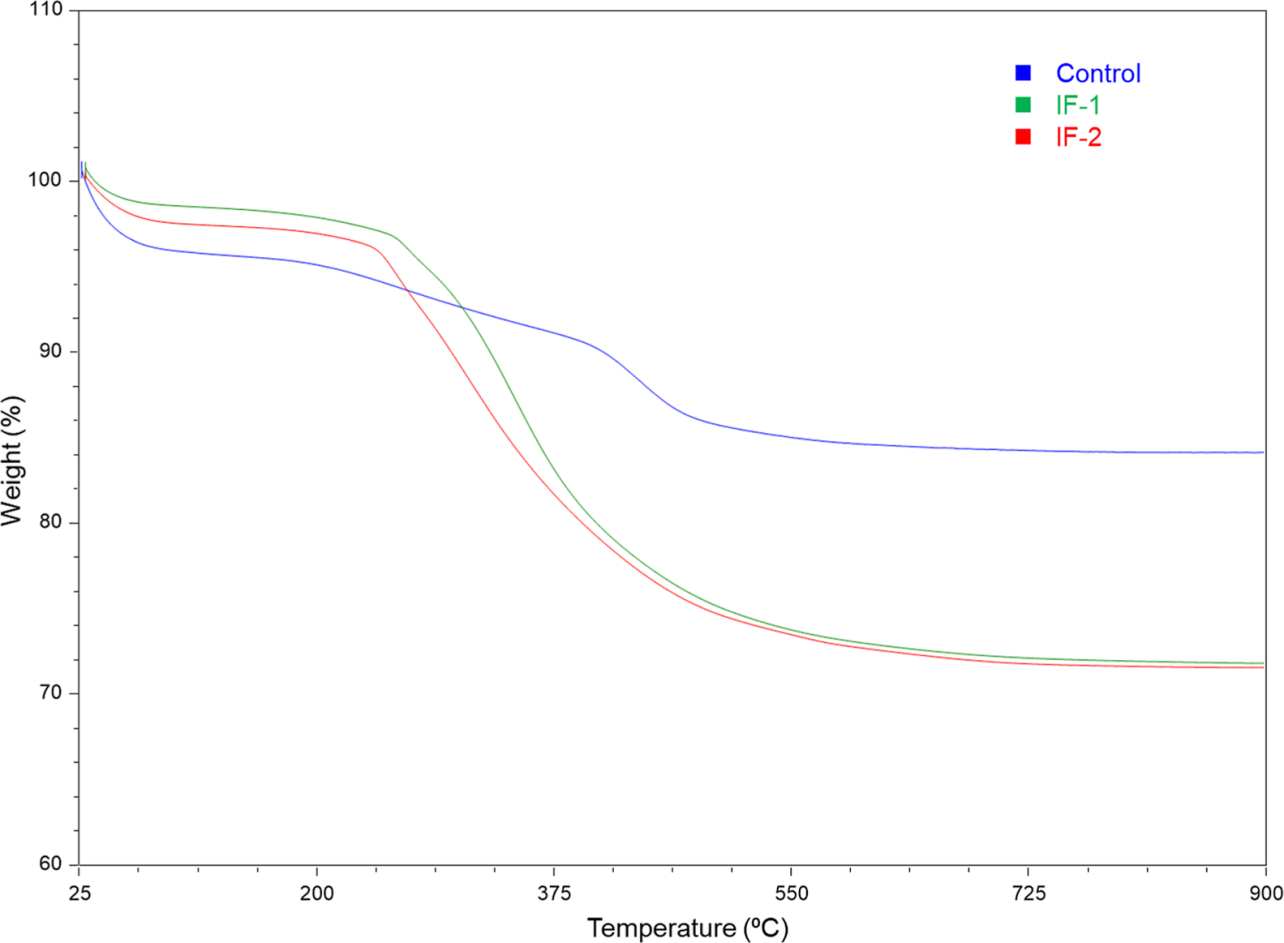
TGA analysis of the control, IF-1, and IF-2. Analysis was performed under N_2_ and the temperature ramped at 10 °C/min from ambient up to 900 °C.

The control sample showed a further mass loss above 200 °C, which accounted for about 11%. This mass loss was assigned to the loss of surface silanols and/or incomplete hydrolysis and condensation of TEOS.^20^ Both IF-1 and IF-2 had similar mass loss profiles above 200 °C, with IF-1 having a slightly higher onset of degradation, 276 vs 243 °C, respectively. With the mass loss being about the same, 25% above 200 °C, the difference in onset might be attributed to how the *n*-octyl functional group was incorporated into the particles. It is possible that with the co-condensation of *n*-OTMS in IF-1, the functional group is more tightly bound within the silica network, resulting in an increase in the thermal stability.^21^

Although TGA gives some insight into the amount of organic composition of the samples, more analysis is warranted to better understand the chemical composition of the nanoparticles. Thus, dry particle samples and *n*-OTMS were characterized by FT-IR spectroscopy. Figure 2 shows the spectra that were collected between 3800 and 2600 cm^–1^.

**Figure 2:**
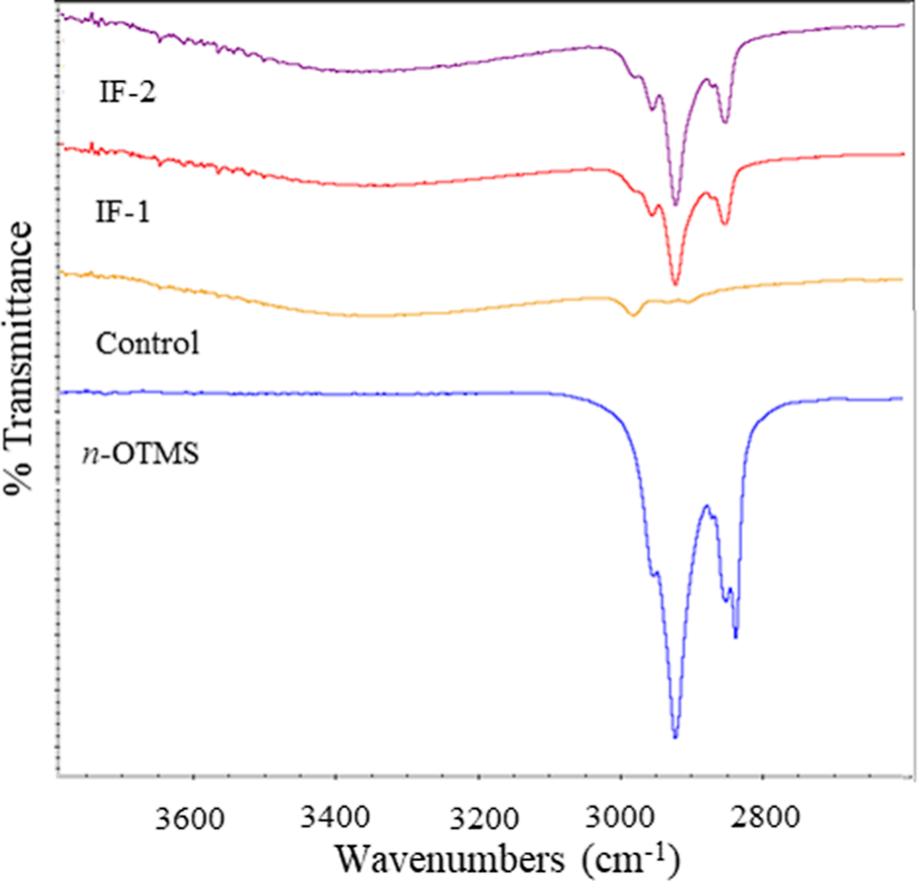
FT-IR spectra from 3800–2600 cm^–1^ of *n*-OTMS, the control, IF-1, and IF-2.

The FT-IR spectrum of *n*-OTMS has noticeable absorption bands between 3000 and 2800 cm^–1^, which were assigned to –CH-stretching. These bands are clearly present in both methods of functionalization. Although less noticeable, there is also weak absorption in this region for the control. Since the control lacks *n*-OTMS, this is likely due to the incomplete hydrolysis and condensation of TEOS and corroborates the TGA data. The control, IF-1, and IF-2 also have a weak and broad absorption band centered at around 3400 cm^–1^, which is assigned to –OH stretching. This absorption band could be due to the presence of physically absorbed water and/or silanols.

Analysis of the fingerprint region for *n*-OTMS, Figure 3, shows an absorption band near 1450 cm^–1^, which was assigned to a –CH-bending mode. Both functionalization methods present similar absorption bands, indicating that the *n*-octyl group was successfully incorporated in the silica nanoparticles. Weak absorption bands in this region are also present in the control, supporting the conclusion that the TEOS did not undergo complete hydrolysis and condensation.

**Figure 3:**
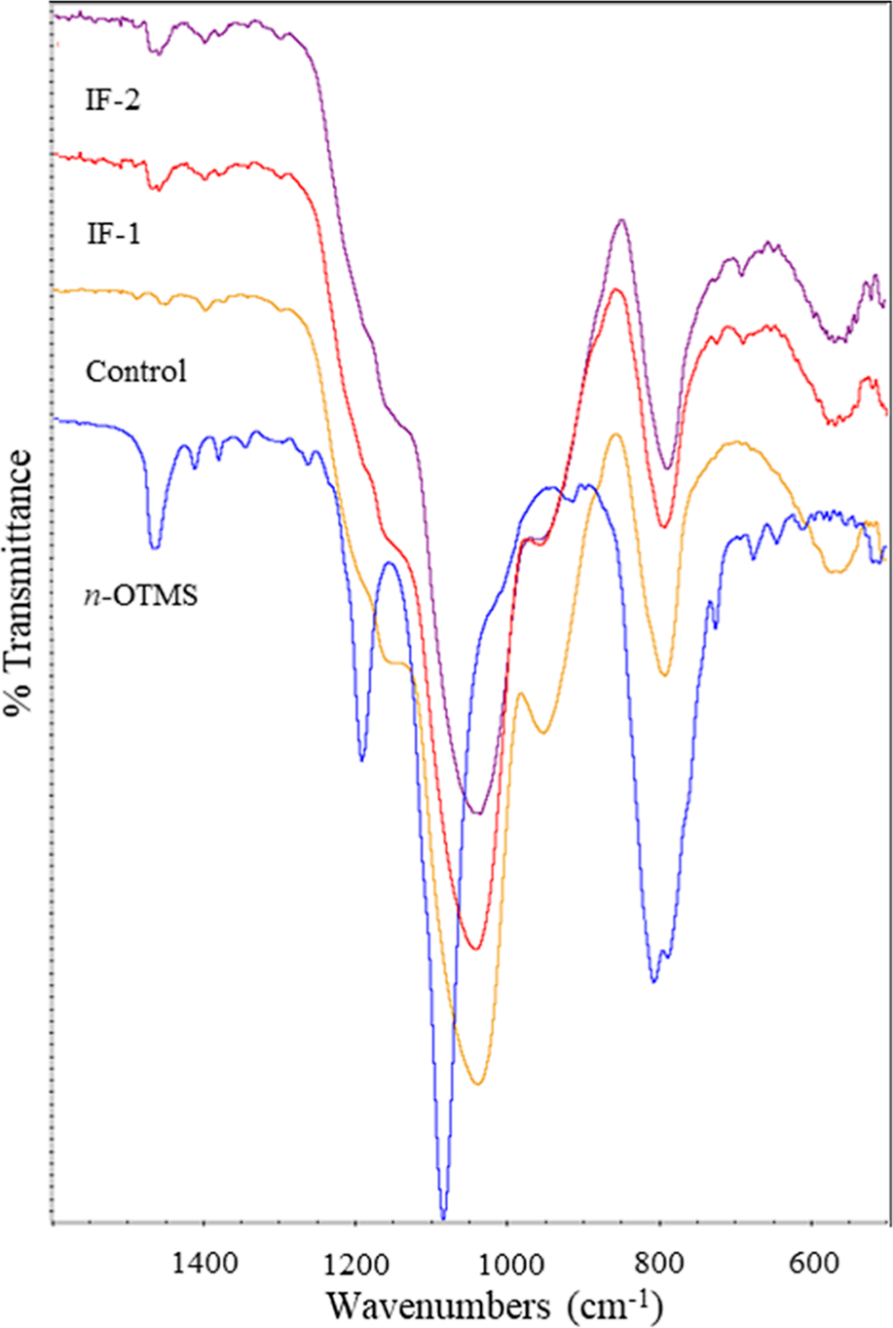
FT-IR spectra from 1600–500 cm^–1^ of *n*-OTMS, the control, IF-1, and IF-2.

All particle samples have strong absorption bands near 1100–1020 cm^–1^ and 800 cm^–1^, which are assigned to Si–O–Si stretching and bending of Si–O, respectively. Absorption bands assigned to the asymmetric bending of Si–OH (950 cm^–1^) are also present in all particle samples and support the presence of silanols. This band is the strongest for the control. The presence of this band and the –CH-stretching and bending modes indicates that the mass loss seen in the TGA data of the control above 200 °C is attributed to both silanols and incompletely hydrolyzed/condensed TEOS. The *n*-OTMS has strong absorption bands between 1190 and 1150 cm^–1^ and at 800 cm^–1^, which are assigned to Si–OCH_3_ stretching and Si–O bending, respectively.^22^

Dry particles were resuspended in absolute ethanol and then characterized by DLS to determine particle size, Figure 4. DLS indicated that the control and IF-1 samples were uniform and had diameters of 504 ± 8 nm and 2000 ± 300 nm, respectively. Although a value was given for IF-2 (∼1800 nm), analysis by DLS also indicated that this sample was too disperse and/or contained significant particle aggregation, preventing an accurate size measurement using this method.

**Figure 4:**
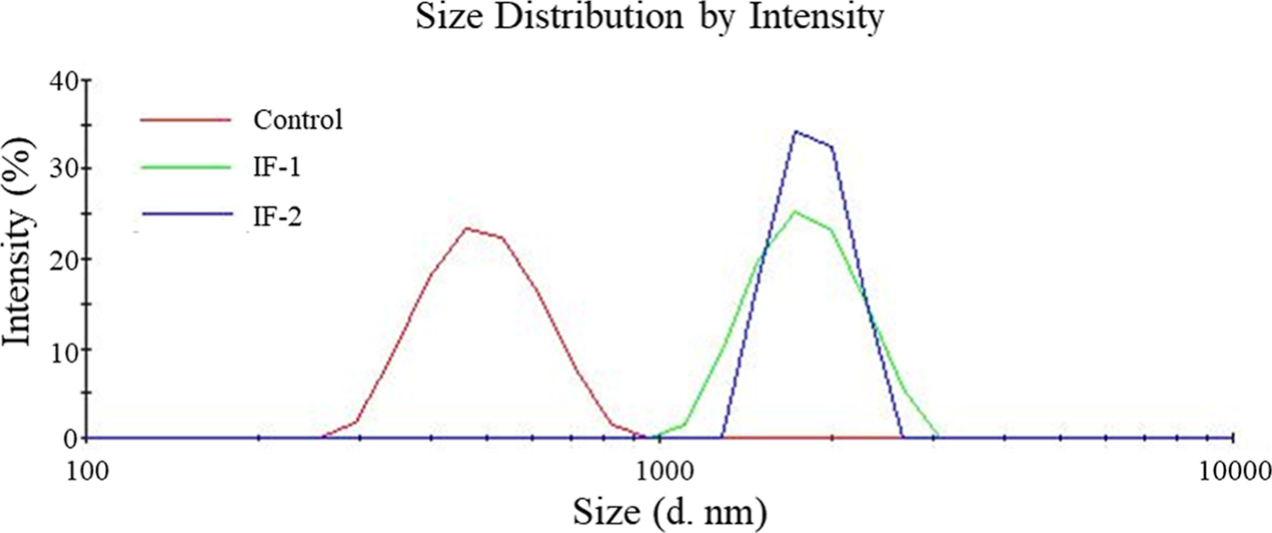
DLS intensity data obtained for the control, IF-1, and IF-2.

Sample morphology of the resuspended particles was initially investigated using SEM, Figure 5. SEM analysis indicated that the control and IF-1 were uniform and monodisperse on the micrometer scale. The diameters were found to be around 480 and 590 nm, respectively. SEM analysis of IF-2 revealed that this sample had a complex morphology, was particle-like, and was not uniform. As with DLS and due to the complexity of the sample, the diameter of IF-2 could not be accurately determined; however, the particulate present appeared to be nanometer sized.

**Figure 5:**
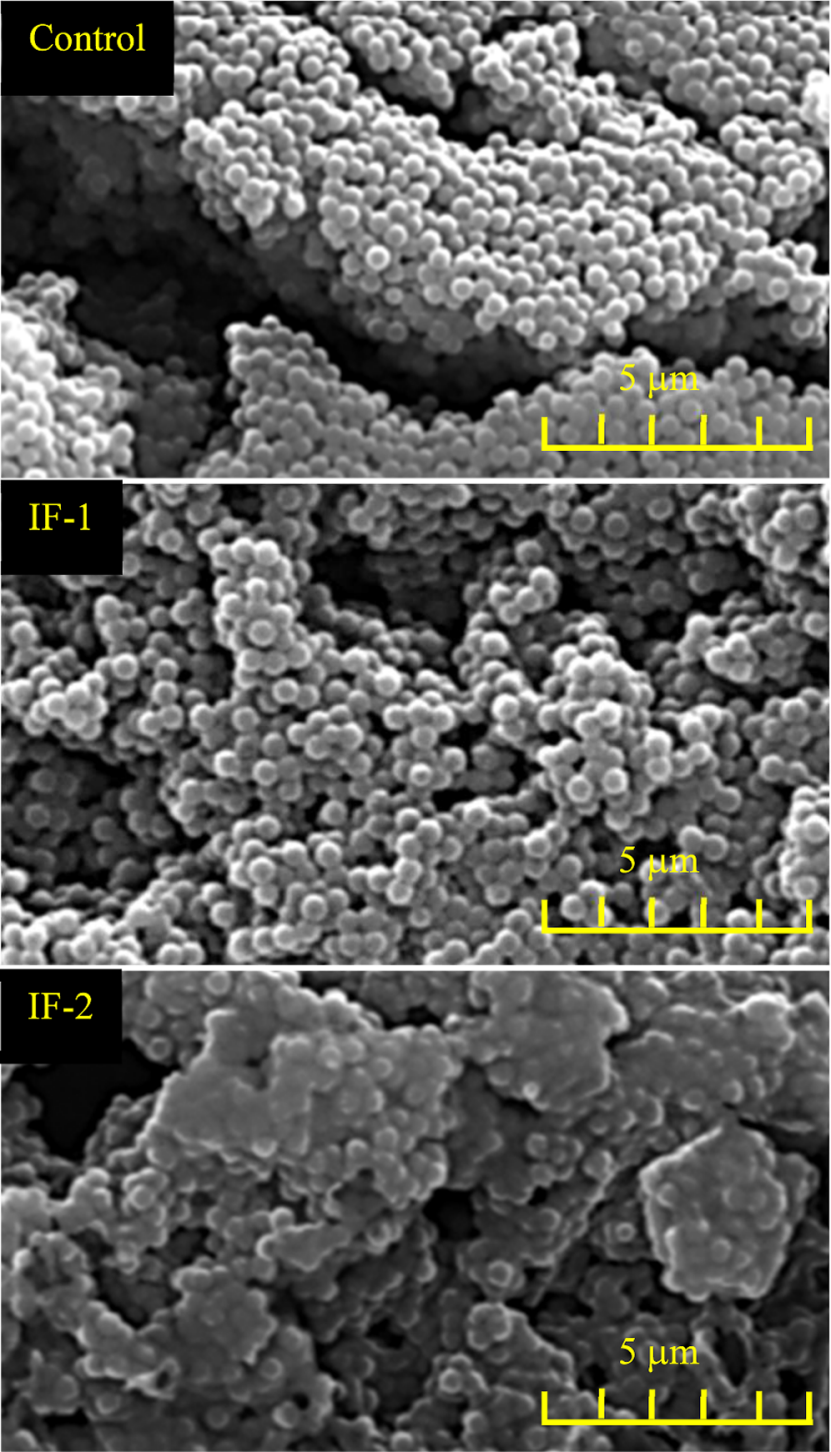
SEM analysis of the control, IF-1, and IF-2.

To further investigate the impact of the functionalization on the surface properties of the particles, glass slides were spin coated with the particle suspensions used in the DLS and SEM analyses. Macroscale surface roughness analysis was performed on each sample using optical profilometry.^23,24^ The average surface roughness of an area, Sa, of the control, IF-1, and IF-2 was found to be 34 ± 6 nm, 30 ± 10 nm, and 23 ± 3 nm, respectively. Furthermore, as shown in Figure 6, the control had a visibly rougher surface at the 800 × 800 μm^2^ scale than both IF-1 and IF-2.

**Figure 6:**
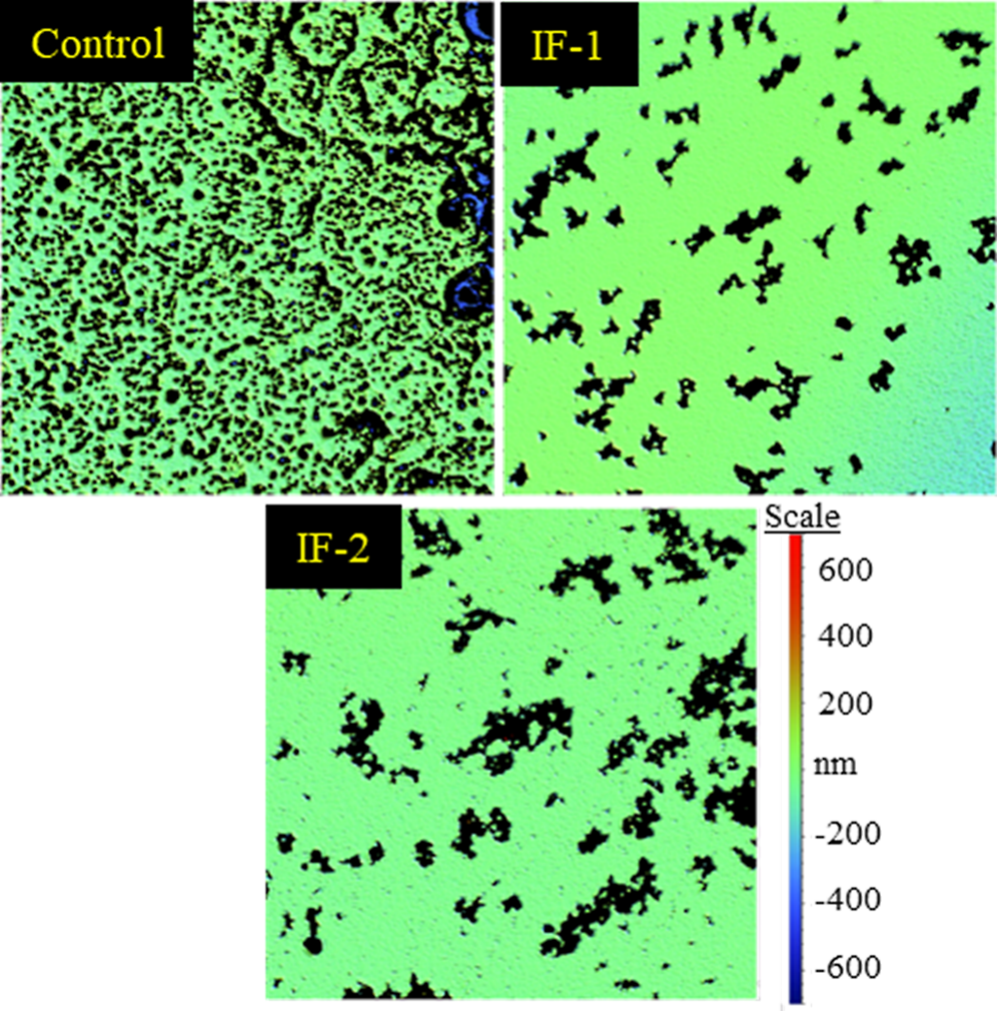
Optical profilometer data for the control, IF-1, and IF-2. Each image is 800 × 800 μm^2^.

AFM was also used to investigate the size and morphology of the nanoparticles as well as the average nanoscale surface roughness, Ra, of the spin-coated surfaces. AFM revealed that the control sample resulted in uniform, spherical nanoparticles, that had a smooth surface, with diameters around 560 nm, Figure 7. Analysis of IF-1 revealed spherical nanoparticles with a mostly smooth surface. Particles were less uniform in size and ranged from about 500 up to 700 nm. The nanoparticles that resulted from IF-2 appeared sphere-like with noticeable roughness on the surface. Due to the roughness and complex morphology of the data collected for IF-2, particles were difficult to size by AFM, however, as with SEM, the particulate present appeared to be nanometer sized. The average surface roughness for the control, IF-1, and IF-2 was found to be 90 ± 10 nm, 225 ± 4 nm, and 265 ± 8 nm, respectively.

**Figure 7:**
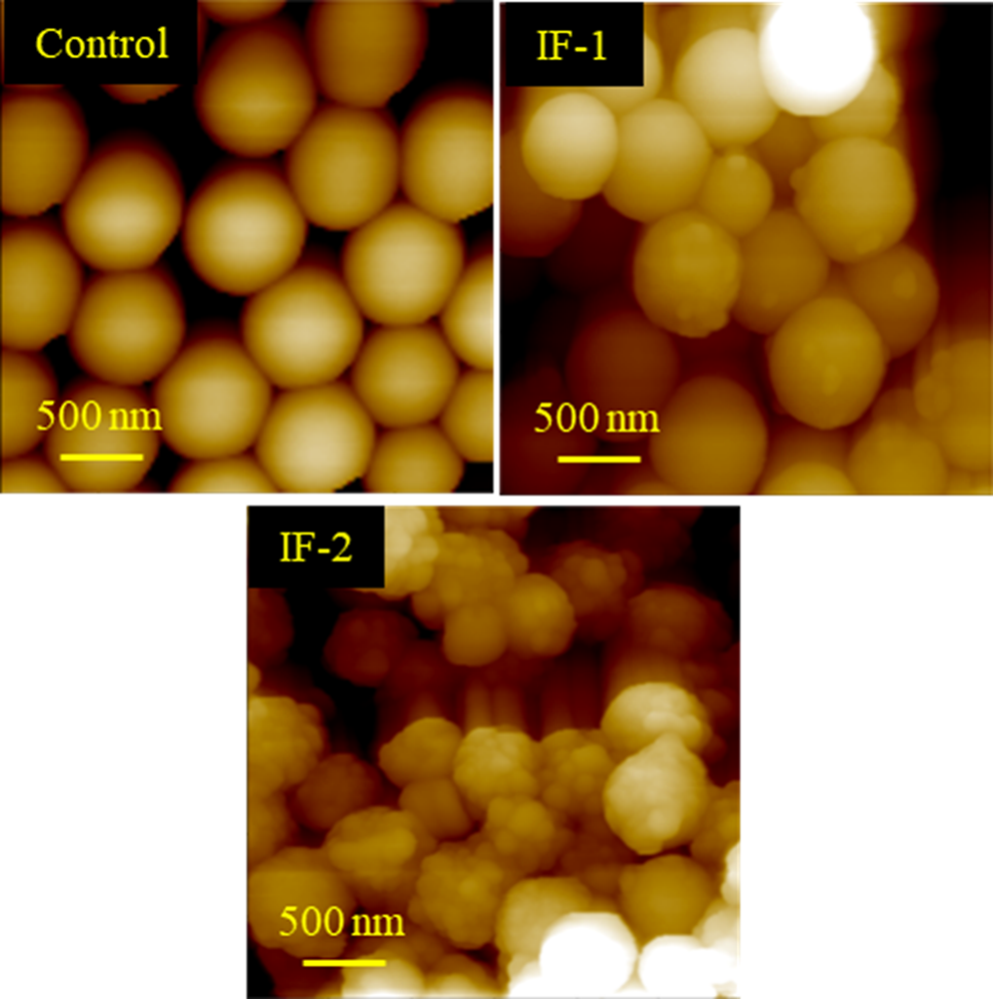
AFM images of the control, IF-1, and IF-2. Each image is 3.0 × 3.0 μm^2^ and was cropped from the original 10 × 10 μm^2^ height images.

WCA analysis was performed on the spin-coated surfaces, Figure 8. The WCA of the control could not be accurately measured as the water completely wet the surface before an image could be taken. The WCA for IF-1 and IF-2 was found to be 67 ± 2° and 86 ± 8°, respectively.

**Figure 8:**
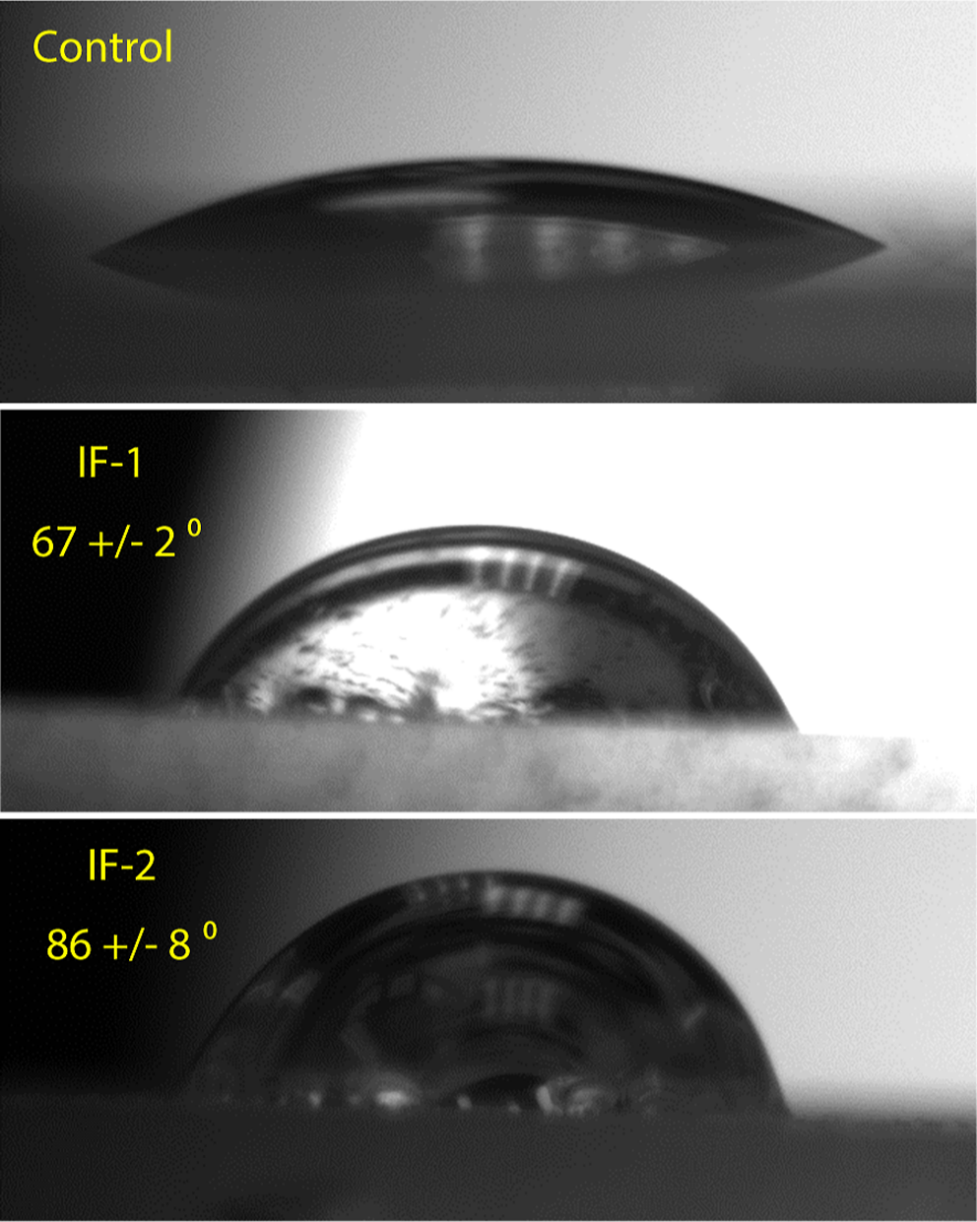
WCA images of the control, IF-1, and IF-2.

Table 2 summarizes the data that were collected by DLS, SEM, AFM, optical profilometry, and WCA analysis. Although the size of the control was within reason for DLS and SEM, less than 5% difference, AFM indicated that the particles were larger. The larger size found by AFM might be attributed to tip broadening from the cantilever.^25^ In looking at IF-1, both SEM and AFM show that the particles were nanometer sized and significantly smaller than what was determined by DLS. With the incorporation of the octyl-hexyl group into the particles, they would be more hydrophobic than the control, which could induce aggregation in ethanol and yield an inflated size by DLS. AFM analysis of IF-1 also demonstrated that this sample was not as uniform in size as what was observed in SEM. The AFM image of IF-1 also indicates that some of the particles had some noticeable surface roughness. Furthermore, although DLS, SEM, or AFM could not adequately estimate the particle size of IF-2, all three methods indicated that this sample was complex, nonuniform, and had visible surface roughness on the particles. Both SEM and AFM analyses of the particles indicate that the method of functionalization also had a noticeable impact on the particle morphology.

**Table 2:** Particle Characterization by DLS, SEM^a^, AFM^b^, Profilometry^c^, and WCA

**sample**	***d* (nm)**	***d*** ^a^ **(nm)**	***d*** ^b^ **(nm)**	**Sa** ^c^ **(nm)**	**Ra** ^b^ **(nm)**	**WCA (** ^**0**^ **)**
control	504 ± 8	480	560	34 ± 6	90 ± 10	^e^
IF-1	2000 ± 300	590	500–700	30 ± 10	225 ± 4	67 ± 2
IF-2	^d^	^d^	^d^	23 ± 3	265 ± 8	86 ± 8

a SEM.

b AFM.

c Profilometry.

d Measurement was not taken due to agglomeration.

e Measurement was not taken due to wetting.

In looking at the average surface roughness of the spin-coated slides, all samples had low surface roughness on the macroscale in comparison to that on the nanoscale.^24^ Optical profilometry indicated that the control had a higher surface roughness, and IF-2 had the least. The opposite trend was observed in AFM. These variations in surface roughness are attributed to the different measurement techniques and resolutions of each characterization method.^26,27^ Optical profilometry utilizes reflected light to generate a 3D map of a surface. Although this usually allows for the measurement of larger areas at faster rates than AFM, the measurement can be impacted more by surface attributes such as reflections or transparency. These factors typically reduce the resolution to the microscale.^28^ AFM on the other hand utilizes a cantilever or tip to scan the surface, line by line, generating a 3D surface map. While this technique results in limits on scanning area and extended sampling times, resolutions on the nanometer scale can easily be achieved.^29^ Furthermore, these results indicate that on the nanoscale, particle morphology plays a more significant role in average surface roughness.^30^

With WCAs being less than 90°, all spin-coated surfaces are hydrophilic, with IF-1 and If-2 being less hydrophilic than the control.^31^ This is attributed to the incorporation of the *n*-octyl group as well as the increased nanoscale surface roughness observed for IF-1 and IF-2.

## Conclusions

This CURE successfully engaged students in the synthesis, functionalization, and characterization of silica nanoparticles by using accessible sol–gel methods. Through systematic comparison of unfunctionalized and *n*-octyltrimethoxysilane-functionalized particles via co-condensation and delayed functionalization, students gained exposure to a range of materials characterization techniques, including FT-IR, TGA, SEM, AFM, optical profilometry, and WCA measurements. The results demonstrated clear differences in particle size, morphology, surface roughness, and hydrophilicity based on the functionalization method, illustrating the impact of the synthetic design on material properties. Importantly, this experiment provided a flexible and scalable platform for introducing fundamental concepts in materials chemistry and nanotechnology within an undergraduate curriculum. The modular design of the laboratory allows for adaptation to a variety of institutional resources and presents numerous opportunities for future exploration and student-driven inquiry.

## Supplementary Material



## Notes

Example student handout, mid-cycle progress review, final written report, and literature article submission (PDF)
