# Supplementary material for: Synthesis and Characterization of Functionalized Silica Particles: A Course-Based Undergraduate Research Experience in Materials Chemistry
Source: ACS Omega. 2025 Oct 3;10(40):47095–102. doi: 10.1021/acsomega.5c05617 (PMC12529174; doi:10.1021/acsomega.5c05617)
Supplement: Supplementary file 1 [file ao5c05617_si_001.pdf]

Supporting Information for Article:

Synthesis and Characterization of Functionalized Silica Particles: A Course-based Undergraduate Research Experience in Materials Chemistry

Marco Bell[1], Elizabeth K. Dierlam[1], Cayden Smith[1], Luke A. Wolf[1, 2], and Abby R. Jennings[1*]

[1]Department of Chemistry, United States Air Force Academy, 2355 Fairchild Drive, USAF Academy, Colorado Springs, CO, USA

[2]Heersink School of Medicine, University of Alabama at Birmingham, 1670 University Blvd, Birmingham, AL, USA

*Corresponding author: abby.jennings@afacademy.af.edu

- Example Student Handout S1-2
- Example Mid-cycle Progress Review S3-4
- Final Written Report S5-30
- Literature Article Submission S31-37

Example Student Handout

NANOMATERIALS – SYNTHESIS, PROCESSING TECHNIQUES, AND CHARACTERIZATION

PREPARATION & EXPERIMENTAL NOTES:

Your notebooks should have all the reagents/solvents listed (*including their structures*) with stoichiometric calculations prepared before you go to lab. Procedures should be transposed into your notebooks as well. Annotate any observations or deviations. We will review operations of the TGA, FT-IR, particle analyzer (DLS), optical profilometer, AFM, and SEM in the lab. Please continue to transcribe instrument parameters into your notebooks.

Synthesis and Functionalization of Silica Nanoparticles (SiNPs)

Initially, prepare a 9M NH_4OH catalyst solution from concentrated NH_4OH (14.5 M). Be sure to prepare enough for each sample of SiNPs. Each group will choose an alkoxysilane to functionalize their SiNPs with ((3-aminopropyl)-trimethoxysilane, *n*-octryltrimethoxysilane, (tridecafluoro-1, 1, 2, 2-tetrahydrooctyl) triethoxysilane, or 3-(acryloxypropyl) trimethoxysilane).

Each group will prepare functionalized particles using the procedures below. NOTE: each group will choose the amount of functionalized silane to add. A control will also be prepared.

In Situ Functionalization 1 (IF1): In a 25 mL Scintillation vial, combine 5 mL of Abs. EtOH and 10 mL of the catalyst solution. After stirring for 5 minutes, add 0.5 mL of TEOS and 0.030 mL - 0.500 mL of your chosen functionalized silane. Vigorously stir the solution until next class period. Your sample should have a nice vortex.

In Situ Functionalization 2 (IF2): In a 25 mL Scintillation vial, combine 5 mL of Abs. EtOH, 10 mL of the catalyst solution, and 0.5 mL of TEOS. Vigorously stir the solution so that the sample has a nice vortex. After stirring for 16 h and with the help of your instructor, the same amount of the functionalized silane as in IF1 will be added to the scintillation vial. The solutions will be vigorously stirred until next class period. Your sample should have a nice vortex.

Control: In a 25 mL Scintillation vial, combine 5 mL of Abs. EtOH and 10 mL of the catalyst solution. After stirring for 5 minutes, add 0.5 mL of TEOS. Vigorously stir the solution until next class period. Your sample should have a nice vortex.

Washing and Drying Procedures for the Silica Nanoparticles

Transfer particle suspensions to pre-weighed centrifuge tubes and centrifuged at 2,500 r.p.m for 10 minutes. The supernatants were discarded, and the pellets were resuspended in 8.0 mL of absolute ethanol. The centrifugation and resuspending of the particles was repeated two more times. The final pellets were placed in a vacuum oven at 85 ^{o}C for 48 hours.

Example Student Handout

Deliverables

Each group will submit a mid-cycle project summary. Summary should include basic procedures/modifications, data summary to date, and suggestions for future work.

Each group will submit the article(s) you used to develop your spin coating method.

Each student will submit their own, original lab report, to include: Title, Introduction, R&D, Conclusions, Experimental and References section.

REFERENCES

W. Stöber, A. Fink and E. Bohn, *J. Colloid Interf. Sci.*, 1968, **26**, 62.
J.-D. Brassard, D. K. Sarkar and J. Perron, *ACS Appl. Mater. Inter.*, 2011, **3**, 3583.
D. A. Keane, J. P. Hanrahan, M. P. Copley, J. D. Holmes and M. A. Morris, *J. Porous Mater.*, 2010, **17**, 145.

Mid-cycle Progress Review Example

I prepared many different silica nanoparticle samples this semester. I used 5 mL Abs. EtOH and 10 mL 9M NH_4OH for created the initial sample base, as well as the constant volume of 0.5 mL TEOS. Measurement for ethanol and ammonium hydroxide were done with graduated cylinders, TEOS with 1 mL disposable syringes, and *n-OTMS* using auto-pipet. I followed the direction pretty closely for everything else. The stir plate was always at 500 rpm and the centrifuge was always at 4000 rpm.

From there, I created samples with variation in: (1) top-down vs. bottom-up functionalization, (2) high and low levels of *n-OTMS*, and (3) amount of time samples were left mixing on the stir plate. The following table contains each sample by name, followed by the volume of *n-OTMS* added and the amount of time spent stirring either before or after adding *n-OTMS*.

1-A	48 hours, No *n-OTMS*
2-A	48 hours, 0.03 mL *n-OTMS* (bottom-up)
2-B	48 hours, 0.03 mL *n-OTMS* (top-down)
3-A	144 hours, No *n-OTMS*
3-B	144 hours, 0.1 mL *n-OTMS* (bottom-up)
3-C	144 hours, 0.1 mL *n-OTMS* (top-down)
4-A	96 hours, 0.1 mL *n-OTMS* hours (bottom-up)
4-B	192, hours, 0.1 mL *n-OTMS* (bottom-up)
5-A	48 hours, 0.1 mL *n-OTMS* (bottom-up)
5-B	19 hours, 0.1 mL *n-OTMS* (top-down)
6-A	48 hours, 0.1 mL *n-OTMS* (top-down)
6-B	24 hours, 0.03 mL *n-OTMS* (top-down)
7-A	48 hours, 0.5 mL *n-OTMS* (bottom-up)
7-B	48 hours, 0.5 mL *n-OTMS* (top-down)
8-A	48 hours, 0.3 mL *n-OTMS* (bottom-up)
8-B	48 hours, 0.3 mL *n-OTMS* (top-down)
9-A	48 hours, 0.4 mL *n-OTMS* (bottom-up)
9-B	48 hours, 0.4 mL *n-OTMS* (top-down)
10-A	48 hours, 0.2 mL *n-OTMS* (bottom-up)
10-B	48 hours, 0.2 mL *n-OTMS* (top-down)

For comparing samples with different amounts of *n-OTMS* added, I would compare 2-A with 5-A, and compare 2-B with 6-A. Samples that used more than 0.1 mL of *n-OTMS* gelled during the washing/centrifuging and were not characterized further.

All samples were inspected using Zetasizer, AFM, and the optical profilometer. Results and pictures of these tests can be found in their respective instruments with files as sample names.

For spin-coating, I used a method that was comprised of 10 seconds at 500 rpm followed by 60 seconds at 2000 rpm. I would just apply one disposable-pipet worth of sample in the first stage of spinning. I would investigate to see if you can program that spin-coater to have lower rpm limits and higher duration limits, which may be useful. The sample seemed to spread most evenly over the surface of the normal microscope slide, no need to use anything with indium.

I used profilometry to examine the uniformity of the surface of the slide. Initial inspections of the microscope slide made me think I was using samples that were too concentrated. So, I took ~0.1-0.3

grams of unknown 3 (to be reidentified later) and suspended it in 10 mL Abs. EtOH in a centrifuge tube. I would mix the solution thoroughly using the automatic vibrator and draw the sample from the middle of the tube, not the bottom. Using this sample, I did 3 spin-coats onto a microscope slide, and they resulted in the best uniformity of anything I have seen so far (according to views through the profilometer).

Recommendations/ Lessons learned:

Moving forward, be sure that if the sample is in solution, you mix it very thoroughly and quickly extract it from the centrifuge tube. I have learned that the nanoparticles do not want to stay suspended in solution, so it may prove difficult to get actual readings of concentration for your results.

To accurately determine the concentration of the solutions I would use for spin-coating, we dried them out in an oven. Remember to label the sample name on both the body and lid of your centrifuge vials (I forgot).

SI

DLS:

	**Control**	**2-A**	**5-A**	**2-B**	**6-A**
**Z-Avg (nm)**	627.5	795.5	1001	935.0	720.9
**Peak Size (nm)**	645.0	818.0	826.9	833.9	270.0
**Intensity (%)**	100	100	100	100	100
**St. Dev (nm)**	122.0	151.6	90.22	89.73	20.46

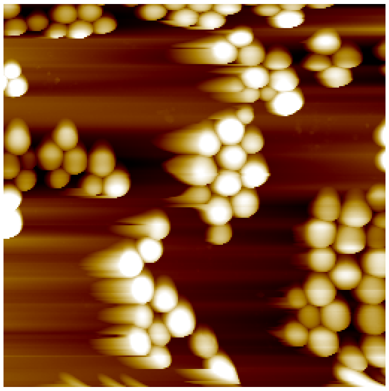

Control (1-A)

Mid-cycle Progress Review Example

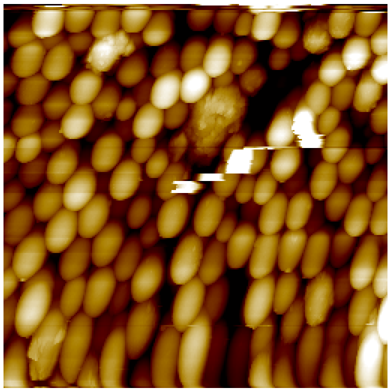

Bottom-Up high (5-A)

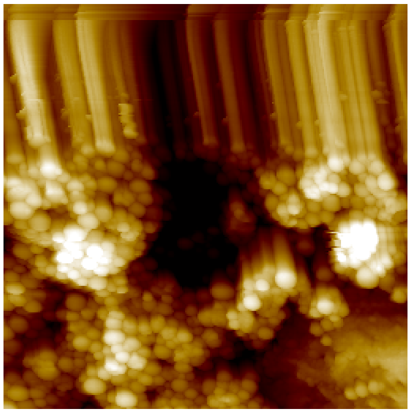

Top-Down High (6-A)

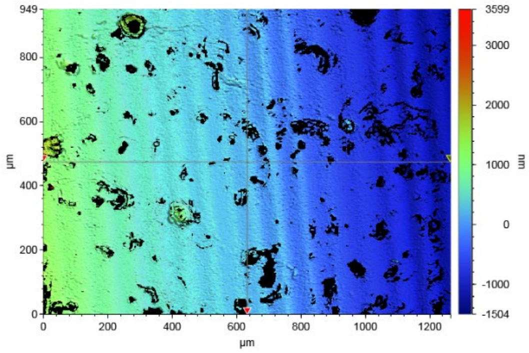

Control (1-A)

Mid-cycle Progress Review Example

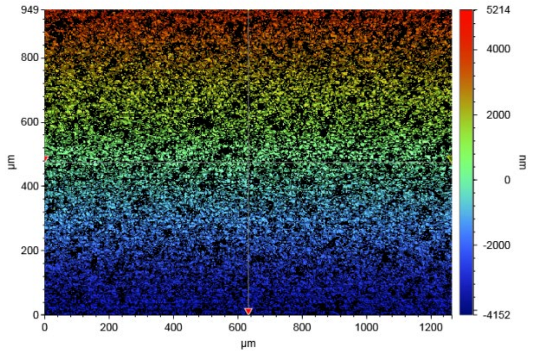

Bottom-Up high (5-A)

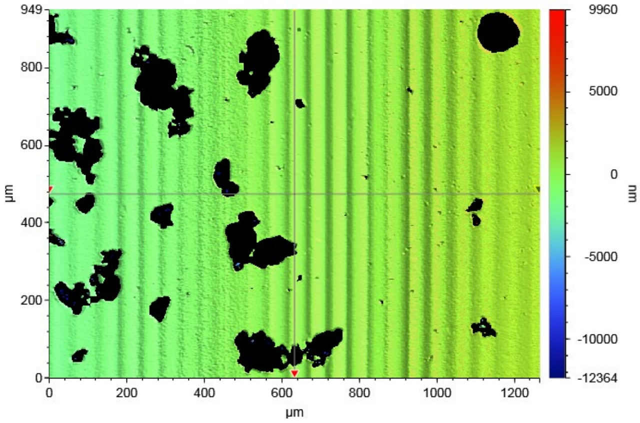

Top-Down High (6-A)

Example Final Written Report

Synthesis, Processing Techniques, and Functional Characterization of Silica Nanoparticles (SiNPs)

ABSTRACT: Herein, silica based nanoparticles were functionalized via a co-condensation and delayed functionalization synthesis. Silicon dioxide (SiO_2) nanoparticles were synthesized via addition of tetraethyl orthasilicate (TEOS) in absolute ethanol and 9M ammonium hydroxide (NH_4OH) catalyst solution. Synthesized SiO_2 nanoparticles contained -OH functional groups capable of subsequent functionalization. In this way, modifying the addition of functionalized silane either immediately (co-condensation functionalization) or after 24 hours (delayed functionalization) would impart specific properties onto the silica nanoparticles, such as particle size and surface roughness. alkyl silane was corroborated by the use of SDT and FT-IR analysis. Characterization of each type of nanoparticle was conducted by scanning electron microscopy (SEM), atomic force microscopy (AFM), solvent contact angle, dynamic light scattering (DLS), simultaneous differential thermal (SDT) analysis, and Fourier trans-infrared spectroscopy (FT-IR). These techniques were leveraged to experimentally determine the impact of functionalization on nanoparticle characteristics and physical properties. When analyzed by SEM, delayed nanoparticles ranged from 186.45 nm to 408.30 nm in size, whereas co-condensation nanoparticles ranged from 280.28 nm to 378.29 nm in size. When analyzed by AFM, delayed nanoparticles ranged in size from 293 nm to 352 nm, whereas co-condensation nanoparticles ranged in size from 293 nm to 508 nm. Finally, when analyzed by DLS for three trials, delayed nanoparticles averaged 365.8 nm, 358.8 nm, and 380.8 nm, whereas co-condensation nanoparticles averaged 482.0 nm, 471.5 nm, and 465.1 nm. Surface roughness measurements by optical profilometry revealed that delayed functionalized nanoparticles had a greater degree of surface roughness than co-condensation functionalized nanoparticles (100.9468

nm v. 82.092 nm), whereas AFM conclude the opposite (80.938 nm v. 102.527 nm). Contact angle measurements on spin coated silicon wafers revealed a higher contact angle for water over n-Hexadecane, revealing that both nanoparticles were hydrophobic.

INTRODUCTION: The development and synthesis of modern nanoparticles equipped with a high degree of functionalization or loading is of prime interest for innovation as they apply to drug loading and subsequent delivery. The ability to equip nanoparticles with functionalized R-groups specific to combating differing types of illnesses therefore elevates their importance in the civilian and military sector. Nanoparticles are classified as zero-dimensional (0-D) structures, due to their similar length and width.[2] Nanoparticles can be functionalized via a delayed or co-condensation functionalization approach. A delayed approach first synthesizes the desired nanoparticle without R-group functionalization, until a desired period has passed. In this case, the bulk material is first synthesized, followed by the desired functionalized nanoparticle.[2] Methods of synthesis include grinding, ball-milling, laser ablation and photolithography.[2] On the other hand, a co-condensation approach synthesizes the desired nanoparticle combined with the functionalized silane at once. In this case, the nanoparticle is synthesized and chemically assembled with the functionalized silane.[2] A 2019 study synthesized fluorinated silica nanoparticles, detailing the co-condensation and delayed functionalization approach as seen in **Figure 1** below.

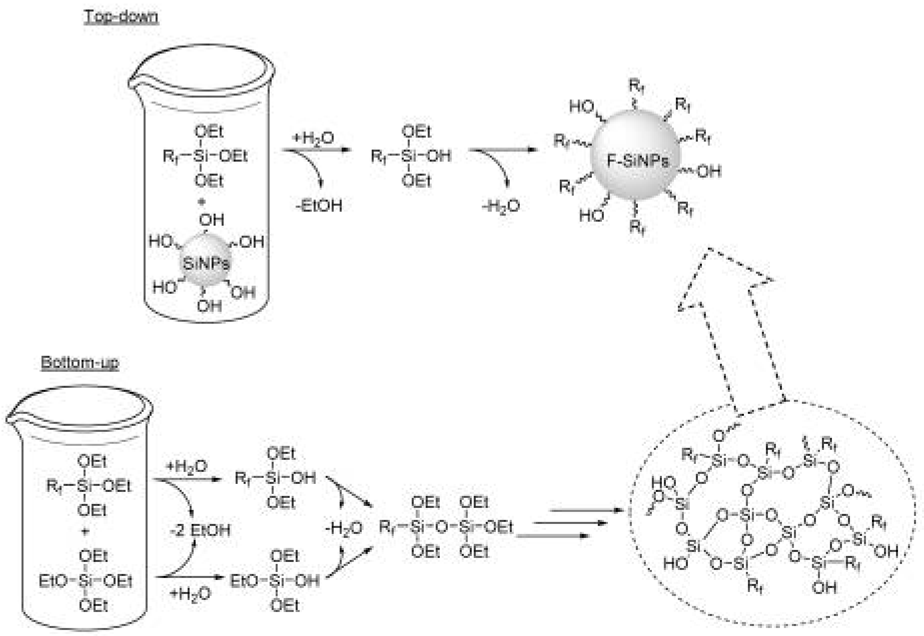

Figure 1. Co-condensation and delayed approach to synthesize fluorinated silica nanoparticles.[3]

The first reported synthesis of a 0-D nanoparticle was in the 1980s, known as the Bucky Ball; laser vaporization and resistance heating (arc evaporation) were leveraged methods to perform the synthesis.[2] One of two different types of 0-D nanoparticles include metallic 0-D structures; methods include laser ablation, laser evaporation, and solution phase, in which a metal salt (ex. CdO, $AgNO_3$, $CuCl_2$), a reducing agent (ex. $NaBH_4$, $Na_3C_6H_5O_7$), and a stabilizer (surfactant, polymer), is used.[2] The other is ceramic and polymeric nanoparticles, synthesized via ball-milling, sol-gel, or emulsions.[2]

The Stöber Method was primarily leveraged for the synthesis and functionalization of nanoparticles reported in this report. The Stöber Method is named after the method proposed by a group of scientists led by Werner Stöber in 1968.[4] The Stöber Method relies on an ammonium catalyzed solution and the condensation of ethoxysilanes, such as TMOS or TEOS, to produce uniform particles.[5] In this case, the addition of the ammonium hydroxide catalyst mediates the

pH level between 11 and 13, and functions as a catalyst.[4] On the other hand, Stöber methods can also be performed in acidic conditions, between a pH of 1 and 4, leveraging, as reported, either HCl or NaF.[5] The advantages of the Stöber Method include the ability to scale up reactions and therefore control certain physical and chemical characteristics of the nanoparticles, including size, shape, porosity and composition.[5] Furthermore, altering and thus optimizing the quality of starting material alters both the chemical and physical properties of nanoparticle.[7] A schematic representation of the Stöber Method is shown below in **Figure 2**.

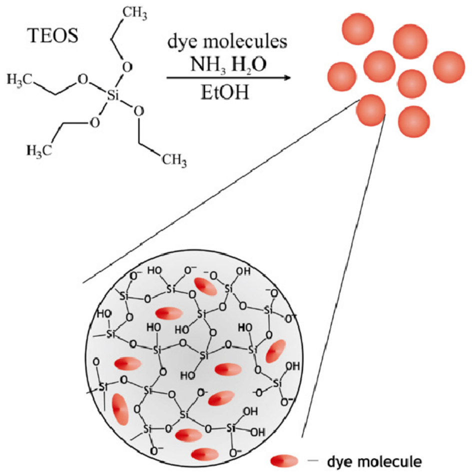

Figure 2. Stöber Method utilized to hydrolyze and condense a silica precursor (dye) in a water/ethanol mixture.[7]

Silica nanoparticles are incredibly versatile and functional particles, due to their ability to introduce what are known as surface functional groups that either interact through the use of an antibody or linker molecule.[7] One source reported modifications including carboxylic acids, primary amines, chloromethyls, (vinylbenzyl) chlorides, hydrazines, azides, aldehydes, thiols and epoxides, however the most common modification to the nanoparticles are organosilanes.[7] The extent to which the physical and chemical properties of two different silica nanoparticles

optimized, not by surface functional group or loading conditions, by time addition of organosilane will be explored, detailed and therefore unlocked in this research paper.

- RESULTS AND DISCUSSION

In order to synthesize the required silicon dioxide nanoparticles, tetraethyl orthasilicate (TEOS) reacted with diluted ammonium hydroxide (NH_4OH) catalyst solution in the presence of absolute ethanol. This reaction produced $Si(OH)_4$, which is shown in the mechanism below cross-reference figures.

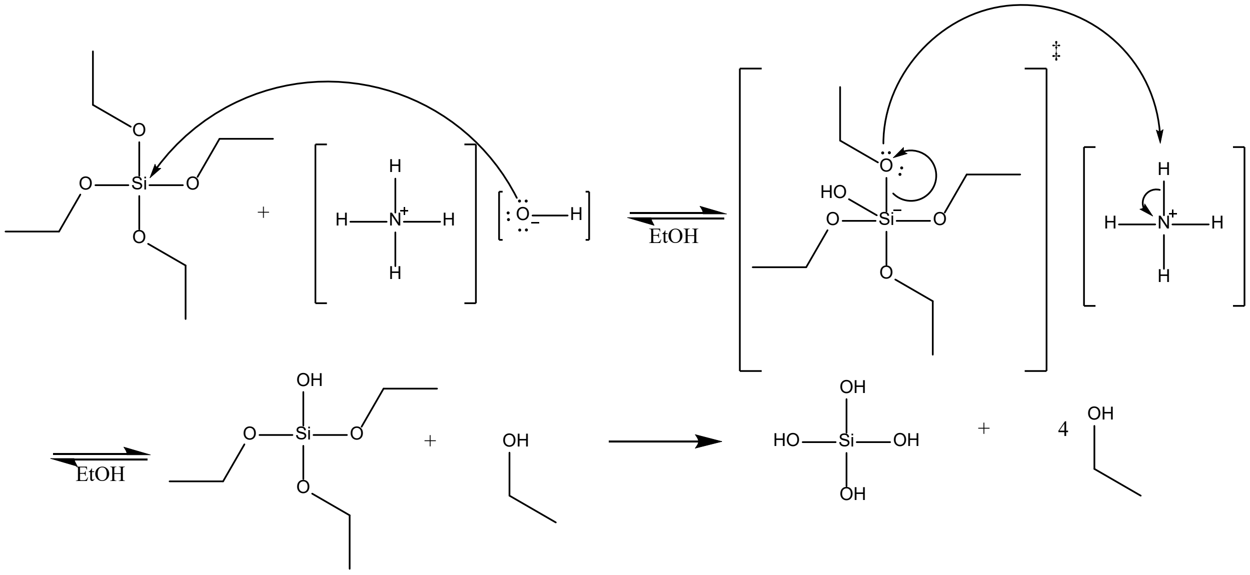

Figure 3. Mechanism for the formation of the silicon tetrahydroxide ($Si(OH)_4$)) precursor.[2]

Reaction of the silicon tetrahydroxide precursor with itself initiates an oligomerization reaction resulting in the formation of water.

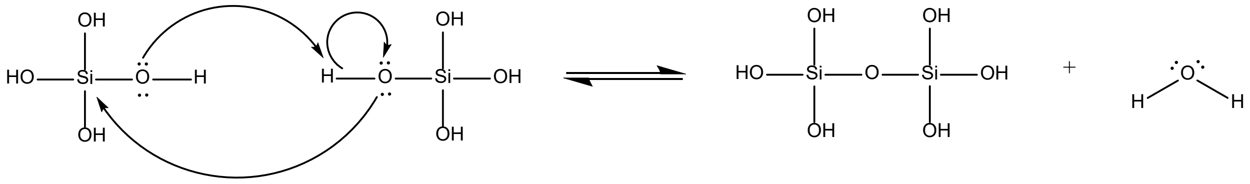

Figure 4. Mechanism for the Si-O bond formation and subsequent oligomerization reaction.[2]

Consequential reaction of $Si(OH)_4$ with the growing reaction chain molecule forms the silicon dioxide nanoparticle. Given the stoichiometry of the reaction itself, the depletion and eventual absence of either ammonium hydroxide or TEOS solution prevents the further addition of $Si(OH)_4$, therefore preventing any further Si-O bond formation. The resulting nanoparticle has hydroxyl functional groups on the surface, as shown below in **Figure 5**, capable of functionalization via a similar chemical mechanism.

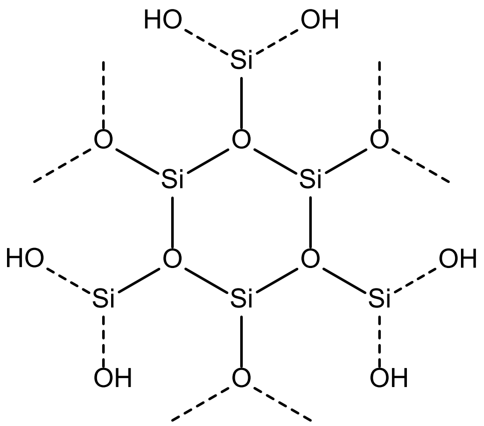

Figure 5. Silicon dioxide nanoparticle[2]

Silica nanoparticles were synthesized and consequently characterized by a variety of methods. Of interest to this experiment was the ability to verify and cross-reference the distribution of sizes of the particles, given that they had been synthesized correctly, in addition to their surface roughness, thermal stability, and physical and chemical composition. Samples of silica nanoparticles, suspended in ethanol via sonication, were drop cast at 3,000 rpm for one minute to obtain a uniform, thin, layer of nanoparticle solution. The uniformity, as determined by? of drop cast nanoparticle solution allowed for more accurate and precise collection of data. Given the

final mass of each particle, and recorded volume of the combined centrifuge tubes, the concentration of nanoparticle solution used for spin coating could be determined.

Table 1. Concentration of Functionalized Nanoparticle Used for Spin Coating

Functionalization	*Mass (mg)	Volume (mL)	Concentration (mg/mL)
**Co-condensation**	154.2	5.00	30.8
**Delayed**	166.4	5.00	33.3

*Mass excludes particles used for the drop cast.

The first set of methods employed in this study were atomic force microscopy and optical profilometry.

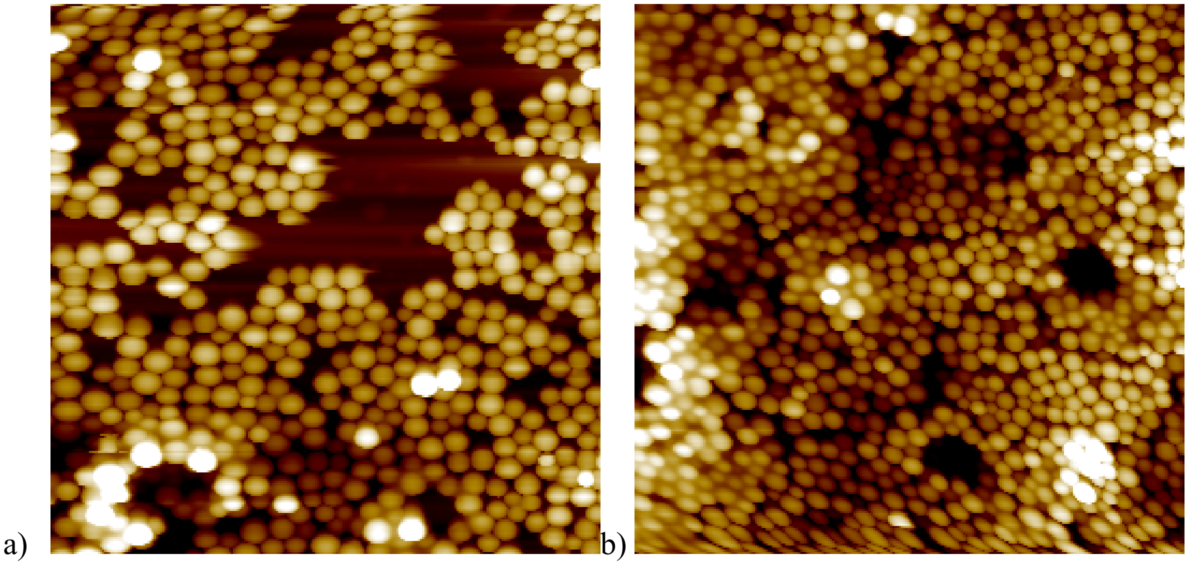

Figure 6. AFM imagery of both the a) co-condensation and b) delayed nanoparticles. Image conditions: a) co-condensation, zoomed-out, height-forward. b) delayed, zoomed-out, height-forward.

AFM imagery revealed greater particle sizes for the co-condensation functionalized nanoparticles, and consequently smaller particle sizes for the delayed functionalized nanoparticles. Moreover, 3D imagery of each nanoparticle sample was captured. Along with quantitative particle size and surface roughness data, 3D imagery obtained qualitative information about the surface roughness, texture and distribution of particles sizes. Altogether, clear resolution of the silica nanoparticles were obtained.

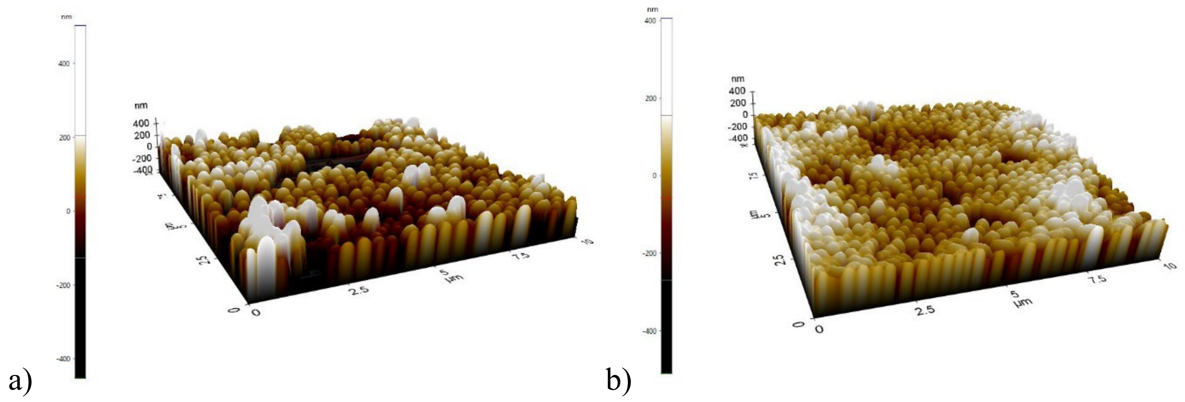

Figure 7. AFM 3D imagery of both the a) co-condensation and b) delayed nanoparticles. Images qualitatively exhibit surface roughness and distribution of particle size.

3D imagery through AFM revealed that the co-condensation functionalization approach yielded larger particles, albeit with a greater distribution in size. On the other hand, delayed functionalization yielded smaller particles that were narrower in the distribution of particle sizes. Broad particle sizes inherent from AFM data revealed that the particles could have been physically compacted and squished together. Altogether, physically seeing and measuring the particles represented a very accurate method to obtain particle size.

d. Results of particle size by AFM are summarized in **Table 2** below. Data is reported as a range between the smallest observed particle, and the largest observed, reported, particle.

Table 2. Particle Size by AFM

Method	Co-condensation (nm)	Delayed (nm)
**AFM***	293.0 to 508.0	293.0 to 352.0

*Particle size data obtained as a range between smallest and largest recorded particle.

Surface roughness was additionally collected for each nanoparticle sample. Results were aggregated below in **Table 3**.

Table 3. AFM Combined Surface Roughness Data

Functionalization	Rpv (nm)	Rq (nm)	Ra (nm)	Rz (nm)	Rsk	Rku
**Co-condensation**	1000.737	124.236	102.527	970.741	0.001	2.775
**Delayed**	915.480	107.849	80.938	909.982	-0.550	4.410

Surface roughness data obtained from height forward/backward microscopy data.

AFM data revealed that the co-condensation functionalized nanoparticles had a greater surface roughness of 102.527 nm, versus 80.938 nm for the delayed functionalized nanoparticles. As previously mentioned, 3D AFM imagery revealed that the co-condensation functionalized nanoparticles had a greater degree of variation. In other words, the presence of larger particles lead to a greater distribution in particle sizes, therefore leading to an increased surface roughness.

The same spin coated silica wafers were analyzed by optical profilometry. Optical profilometry produced data as presented in **Figure 8** below.

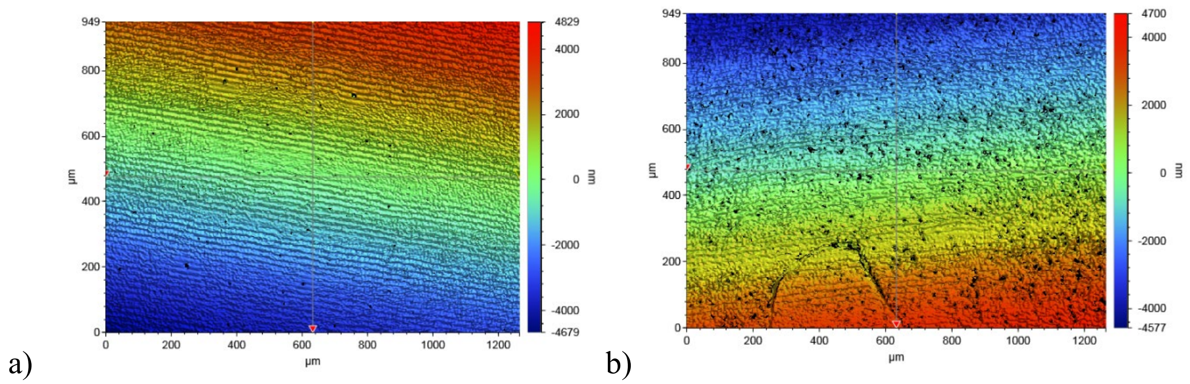

Figure 8. Optical profilometry imagery of both the a) co-condensation and b) delayed nanoparticles. Co-condensation imagery represents trial #3, and delayed imagery represents trial #4.

Imagery initially revealed that the delayed functionalization approach would yield a larger value for surface roughness, due to the presence of rough surface particles found in **Figure 8b**, compared to **Figure 8a**. Combined quantitative date for optical profilometry was compiled into **Table 4** below.

Table 4. Optical Profilometry Combined Surface Roughness Data

		**1**	**2**	**3**	**4**	**5**	**Average**
**Co-condensation**	Sa (nm)	92.577	65.576	83.727	90.061	78.519	82.092
	Sku	7.779	19.137	10.214	6.851	2.931	9.3824
	Sp (nm)	513.605	536.767	664.24	823.85	419.275	591.5474
	Sq (nm)	116.173	83.523	107.759	116.136	98.259	104.37

	SSk	-0.263	-0.722	-0.484	-0.302	-0.02	-0.3582
	Sv (nm)	-2467.14	-2218.89	-2379.93	-2176.66	-727.775	-1994.08
	Sz (nm)	2980.74	2755.655	2044.17	3000.512	1147.05	2385.625
**Delayed**	Sa (nm)	86.957	87.418	100.953	125.64	103.766	100.9468
	Sku	3.247	7.961	3.947	5.357	3.696	4.8416
	Sp (nm)	540.59	2424.366	1274.942	851.361	940.955	1206.443
	Sq (nm)	108.712	114.255	129.498	159.881	133.021	129.0734
	SSk	-0.138	0.379	-0.199	-0.517	-0.25	-0.145
	Sv (nm)	-761.32	-657.187	-804.606	-2537.64	-846.029	-1121.36
	Sz (nm)	1301.91	3081.553	2079.548	3389.005	1786.984	2327.8

Conditions: speed 1x, backscan 15 μm, length 15 μm, threshold 5%.

Of interest to optical profilometry is the average surface roughness (Sa) and surface roughness root mean square (Sq). Evidently, the average surface roughness of the co-condensation nanoparticles was less than the average surface roughness of the delayed nanoparticles. The fluorinated silane was not added to the delayed sample until 24 hours had passed, whereas it was added almost immediately to the co-condensation sample. Given that the silane was added much

later than the other starting materials for the delayed sample, it is likely that the silane could bond along the surface of the silicon dioxide nanoparticles. On the other hand, though R-groups connected to the silicon atom in the silane do not like to be within the nanoparticle, and mainly on the surface, it is more likely that immediate addition of the silane would prevent the build-up of groups attached to the surface of the nanoparticle, thus leading to and corroborating the lower average surface roughness for the co-condensation nanoparticles. Importantly to note, optical profilometry represents an optical measurement of surface roughness, which represents a less accurate method when compared to AFM, which interacts with the surface. In this case, AFM surface roughness data should be accepted.

The spin coated nanoparticle wafers were then tested by solvent contact angle; these tests were performed last of the three due to destruction of the spin coated surface. To the surface of the co-condensation and delayed functionalized nanoparticles, a drop of 18-milli Q water and n-Hexadecane was added. Using the imagery capabilities from an iPhone XR, and a protractor app, the solvent contact angle could be determined, and the hydrophobicity of the nanoparticles could be successfully determined.

a) 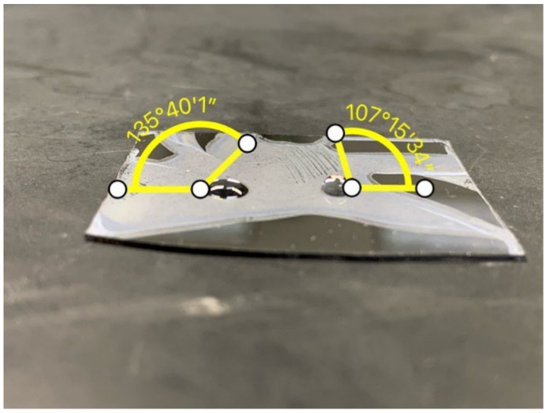

b) 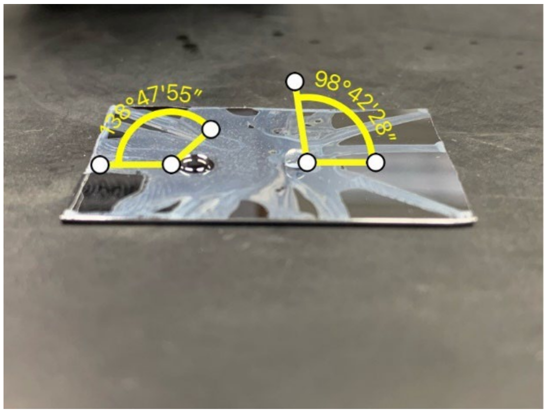

Figure 9. Solvent contact angle testing of both the a) co-condensation and b) delayed nanoparticle spin coated wafers. n-Hexadecane shown as left droplet, and 18-milli Q water shown as right droplet.

Results of solvent contact angle concluded that the contact angle with n-Hexadecane for the co-condensation and delayed functionalized nanoparticle solution was roughly 135° and 138°, respectively. With respect to water, roughly 107° and 98°, respectively. In conclusion, both samples were hydrophobic. The water did not spread out as much onto the surface of the sample, it was being repelled, and therefore preferred to interact with the non-polar solvent.

Dynamic light scattering (DLS), also known as a particle sizer, was leveraged to corroborate particle size and to determine its precision compared with more accurate means of determining particle size, such as AFM or SEM. DLS results were shown as a gaussian distribution of particles, with the average particle size across each trial reported. Distribution profiles of each sample is shown below in **Figure 10**.

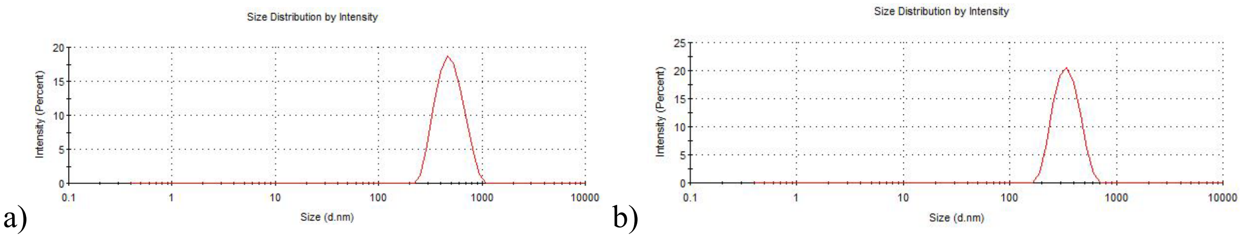

Figure 10. DLS distribution of particle sizes for a) co-condensation and b) delayed nanoparticles.

Quantitative particle size data as an average across the three trials is reported below in **Table 5**.

Table 5. Particle Size by DLS

Method	Co-condensation (nm)	Delayed (nm)
**DLS**^	482.0, 471.5, 465.1	365.8, 358.8, 380.8

^Particle size data obtained as an average across three trials.

DLS reported larger particle sizes from the co-condensation functionalized particles, over the delayed functionalized particles. In line with AFM data, the results are corroborated for similar reasons in terms of R-group functionality based upon addition/functionalization method. However, in terms of accuracy, AFM data should be accepted as more accurate in terms of what could be seen, modified and recorded by eye, rather than light scattering of a particle. It should be noted that the average sizes recorded by DLS fall within the range measured by AFM. I think this means that the DLS data is good too,,,Scanning electron microscopy (SEM) was conducted along with energy dispersive x-ray (EDX) analysis..

a) b)

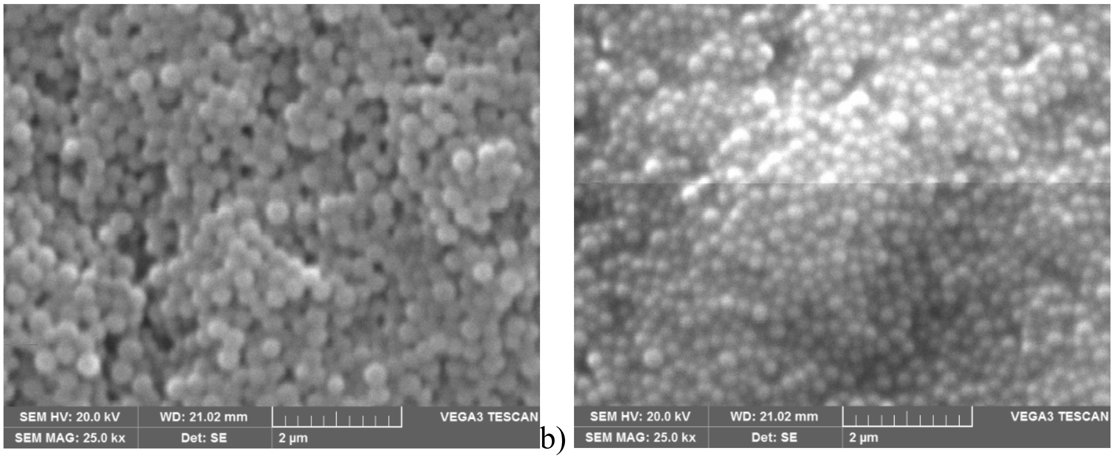

Figure 11. SEM images of the a) co-condensation and b) delayed functionalized nanoparticles. Image conditions: a) resolution, speed 6, MAG 25kx, WD 21.02. b) resolution, speed 7, MAG 25kx, WD 21.02.

At the same magnification and working distance, it is clear that the co-condensation nanoparticles are much larger in size and distribution. For a third time, the larger particle size of

the co-condensation particles, over the delayed particles, is corroborated. Instrumental analysis of the particles yielded the following SEM particle size data, presented as an aggregate of the three particle size methods below in **Table 6**.

Table 6. Overview of Particle Size by Collection Method

Method	Co-condensation (nm)	Delayed (nm)
**AFM***	293.0 to 508.0	293.0 to 352.0
**SEM***	280.28 to 378.29	186.45 to 408.30
**DLS^**	482.0, 471.5, 465.1	365.8, 358.8, 380.8

*Particle size data obtained as a range between smallest and largest recorded particle. ^Particle size data obtained as an average across three trials.

Each nanoparticle sample was studied for its thermal characteristics, in order to understand the functionalization of the nanoparticle upon addition of fluorinated silane. In this way, the mass loss would lead to an understanding of the R-group attachment to the OH functional groups.

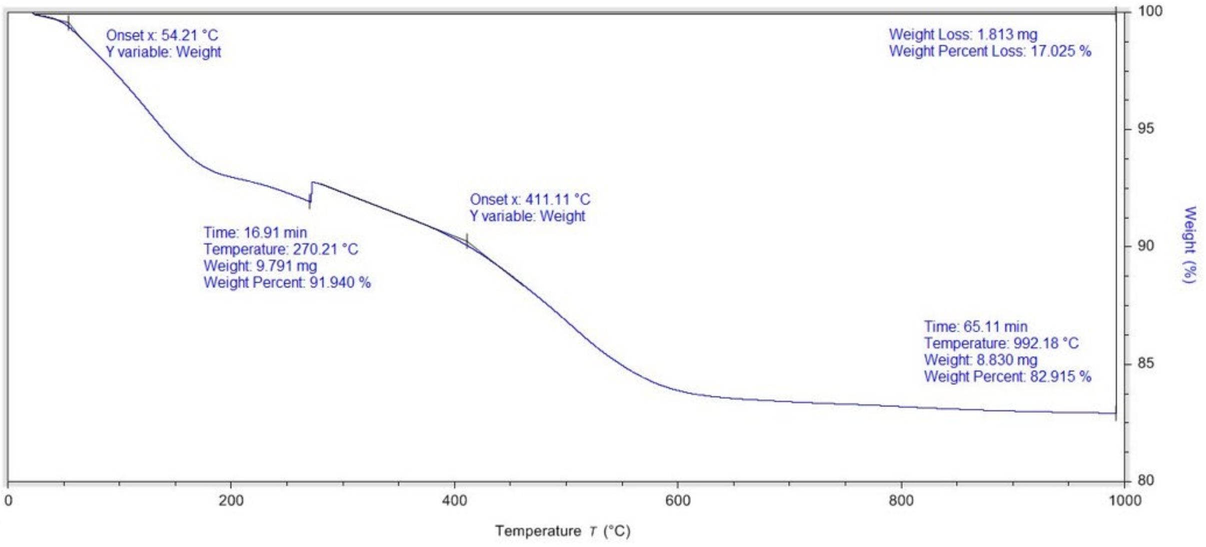

a)

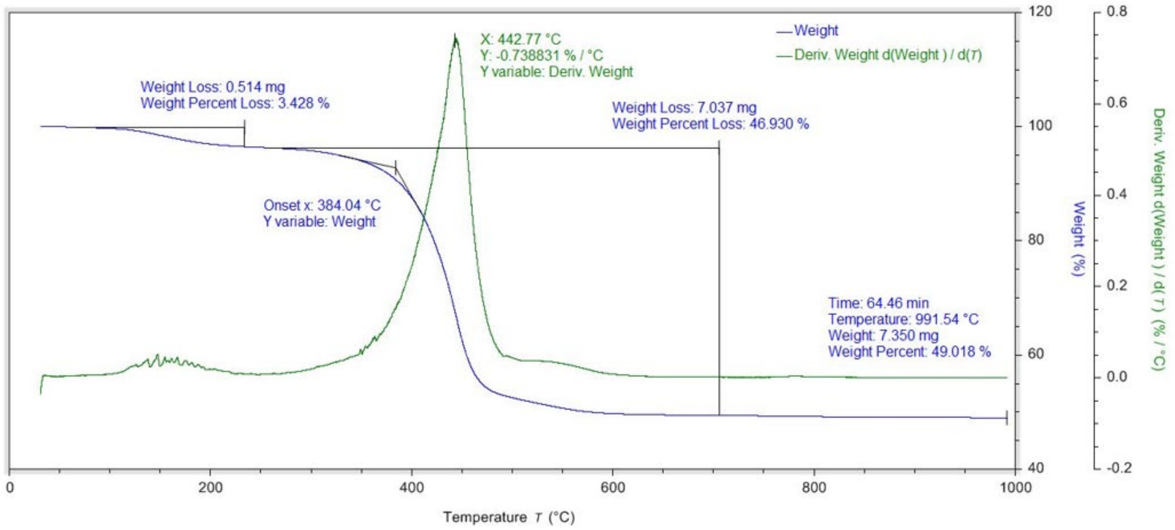

b)

Figure 13. SDT analysis of the a) co-condensation and b) delayed functionalized nanoparticles. Conditions: ambient to 1000 °C, ramp of 15 °C min^{-1}, under N_2. Degradation temperatures reported at the onset of weight loss.

The delayed nanoparticles contained a considerable amount of volatiles, which amounted to roughly 3.428 weight percent of the nanoparticle starting mass. On the other hand, the co-condensation nanoparticles did not display a considerable loss of mass due to volatiles, rather, mass loss was immediate during the course of the ramp. Of interest, however, as shown in **Figure 13**, the co-condensation functionalized nanoparticles retained roughly 82.915 weight percent of initial mass following the ramp to 1000 °C, whereas the delayed functionalized nanoparticles retained only 49.018 weight percent of initial nanoparticle; by excluding potential volatile content, that number jumps up to 52.446 weight percent. Analysis of a control sample of silicon dioxide nanoparticles, without functionalization, revealed a weight loss of 4.839 percent, attributable to volatile content. In this case, the co-condensation nanoparticles are likely to have retained 87.754 weight percent of their initial mass. Given that mass loss is correlated with functionalization, it is clear that the delayed nanoparticles had considerably better

functionalization and subsequent attachment to the OH functional groups on the silicon dioxide nanoparticle.

FT-IR was leveraged to determine the functionalization of each nanoparticle sample. Additionally, a control was run which did not contain any functionalized silane.

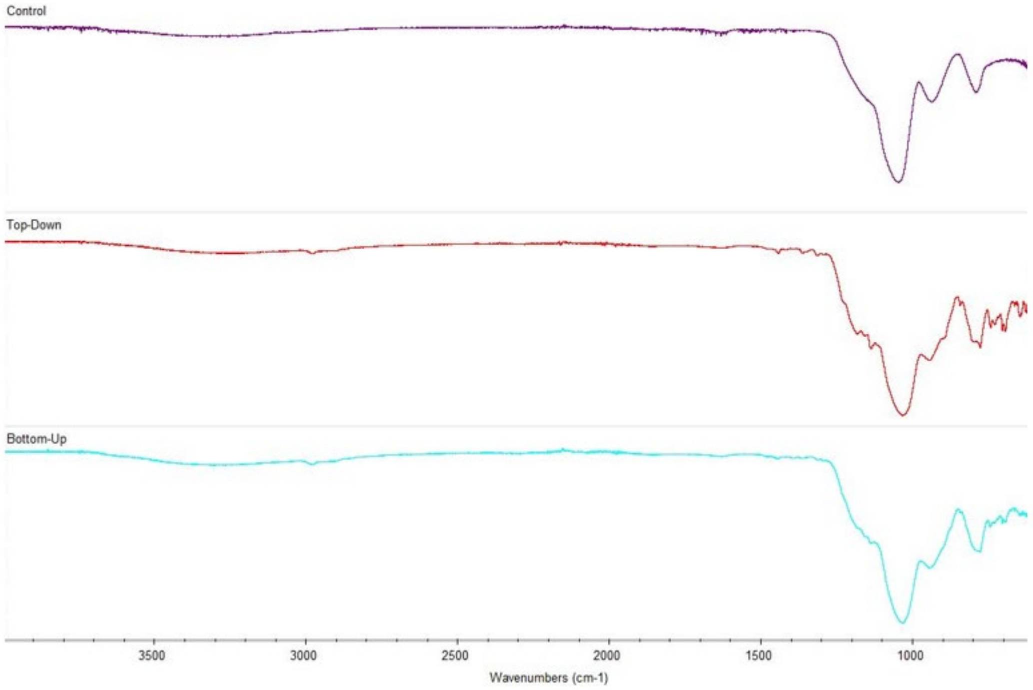

Figure 15. FT-IR overlay spectrum of a silicon dioxide control nanoparticle and two functionalized nanoparticles (delayed and co-condensation).

Signal intensity below 500 cm^{-1} is evidence that functionalization had occurred on the surface of both functionalized silica nanoparticle. The silicon-oxide peak at 1050 cm^{-1} masks further functionalization of the nanoparticles, due to its broad intensity. It's very small, but it looks like there is CH stretching too in the BU and TD. This would be from the R group.

- CONCLUSIONS

Silica based nanoparticles were functionalized via a co-condensation and delayed functionalization synthesis approach. Silicon dioxide (SiO_2) nanoparticles were synthesized via addition of tetraethyl orthasilicate (TEOS) in absolute ethanol and 9M ammonium hydroxide (NH_4OH) catalyst solution. Synthesized silicon dioxide nanoparticles contained OH functional groups capable of subsequent functionalization.

In summary, there is striking difference in the characteristics of each functionalized silica nanoparticle. A co-condensation approach to the addition of functionalized silane yielded larger nanoparticles, with greater surface roughness, and less functionalization silane, when analyzed by AFM, SEM and DLS, and SDT, respectively, as compared to a delayed approach. Incorporation of silane within and onto the surface of synthesized silicon dioxide nanoparticles was observed and hence corroborated through FT-IR, and SDT analysis. SDT analysis was primarily leveraged to draw conclusions for functionalization, based off the degree of weight loss for an entire sample of nanoparticle. The ability to view the surface of the silica nanoparticles was done via SEM and AFM; these methods were accepted over DLS to determine particle size.

Further and future experimentation with functionalized silica nanoparticles should focus on continued experimentation with SEM. SEM particle size analysis should be conducted over a wide range of areas, in hopes to collect a wider range of particle sizes. The synthesis, characterization and analysis of nanoparticles brought valuable insight into the potential applications and implementations of this type of research. Previous experience and experimentation with nanoparticles has informed of their drug delivery capabilities, however, modifying their functionalization therefore represents a potential advancement of either drug delivery or photodynamic therapy (PDT). Altogether, each component of the experiment performed in line as initially expected, providing data that made sense and could be integrated

and cross-referenced with other analysis done during the course of the experiment. For this reason, the experiment was an enjoyable process and very meaningful learning experience.

- EXPERIMENTAL SECTION

Chemicals. Tetraethyl orthosilicate (TEOS) was purchased from TCI America and used as received. (Tridecafluoro-1, 1, 2, 2-tetrahydrooctyl) triethoxysilane orthosilicate ($SiC_{14}H_{19}F_{13}O_3$) was purchased from Gelest INC and used as received. Ammonium hydroxide (NH_4OH) catalyst solution was purchased as a concentrated standard (14.5 M) from Acros Organics and prepared as a 9M stock solution via dilution in 18 milla-Q water. Absolute ethanol (Abs. EtOH) was purchased from Acros Organics and used as received. n-Hexadecane (99%, pure) was purchased from Acros Organics and used as received.

Synthesis of Silica Nanoparticles

Delayed functionalization: To a 25 mL scintillation vial was added 5.15 mL of Abs. EtOH, 10.25 mL of a 9M NH_4OH catalyst solution, 0.5 mL of TEOS and a stir bar. The solution was capped and vigorously stirred for 24 hours. After 24 hours, 0.100 mL of (tridecafluoro-1, 1, 2, 2-tetrahydrooctyl) triethoxysilane orthosilicate was added. The vial was capped and left to stir for an additional 24 hours.

Co-condensation functionalization: To a 25 mL scintillation vial was added 5.15 mL of Abs. EtOH and 10.25 mL of a 9M NH_4OH catalyst solution, along with a magnetic stir bar. The solution was vigorously stirred for five minutes. Following, 0.5 mL of TEOS and 0.100 mL of (tridecafluoro-1, 1, 2, 2-tetrahydrooctyl) triethoxysilane orthosilicate was added. The vial was capped and left to stir for 48 hours.

Washing and Drying of Silica Nanoparticles

Each nanoparticle sample was divided between two centrifuge tubes, so that an equal volume of sample was added to each; roughly 8.0 mL of sample was added to each tube, totaling four tubes. The samples were centrifuged for 15 minutes at 4000 rpm. The supernatant was pipetted out and discarded, and the pellet/particles were re-suspended in Abs. EtOH (5.0 mL). The subsequent washing and re-suspension of the nanoparticles in EtOH was repeated twice more. After the last centrifuge and removal of the supernatant, each pellet was re-suspended in Abs. EtOH (2.5 mL) and each sample was combined into a single centrifuge tube (one delayed and one co-condensation). The final amount of solvent was documented to determine concentration. Following the necessary characterization and analysis on the suspensions, each sample was centrifuged and the supernatant was removed. The tubes were added to a vacuum oven for 48 hours. The dried particles were added to separate, pre-weighed, 25 mL scintillation vials yielding delayed (0.1664 g) and co-condensation (0.1542 g) silica nanoparticles.

Characterization.

Simultaneous differential thermal analysis (SDT) data of each silica nanoparticle was obtained using a TA instrument SDT650, equipped with an autosampler. The samples were heated from ambient conditions to 1000 °C, with a ramp rate of 15 °C min^{-1}, under N_2. All degradation temperatures (T_d) were obtained from the onset of weight loss; themograms or themal data were analyzed using Trios software. Fourier transform infrared (FT-IR) spectra were obtained on a ThermoFisher Scientific Nicolet iS10 FT-IR spectrometer; a background was run before the analysis of each sample. Data was collected in %Transmittance, after 16 scans, with 0.482 cm^{-1} data spacing, and analyzed using OMNIC software. Scanning electron microscope (SEM) images were captured via a Metek scanning electron microscope, under vacuum, equipped with an energy dispersive x-ray (EDX) spectrometer; image analysis was completed with VegaTC

software. Atomic force microscopy (AFM) imagery was collected using a Park Systems NX10 Atomic Force Microscope. Images were collected at 512 pixels and a scan size of 10 µm, and analyzed using XEI software. All composite samples were coated with a thin layer of gold, via a Quorum Electron Microscopy Science (EMS) sputter coater. Optical profilometry imagery was obtained on a 6650P Contour GTK 3D Laser Surface Microscope, with analysis done using Vision64 software. Dynamic light scattering (DLS) particle size data was obtained on a Malvern Zetasizer Nano – ZS Series Particle Sizer, with analysis done using Zetasizer software. Silica nanoparticles were spin coated with a MTI Corporation VTC-100100 Vacuum Spin Coater. Samples were spin coated with ~20 drops of SiNP solution at a spin rate of 3,000 rpm, for one minute. Each silica nanoparticle solution was centrifuged using a Cole Parmer VS-4000 Centrifuge. Samples were centrifuged at a spin rate of 4,000 rpm, for 15 minutes.

REFERENCES

1) Jieyu Zhang.; Jingjing Min.; Binghan Li.; Wenxing Yang.; Zaiping Zeng.; Dianyi Liu.; Botao Ji. *Chemistry of Materials* **2023** 35 (3), 1325-1334
2) Jennings. Abby, R. Nanomaterials Lab – Synthesis, Processing Techniques, and Characterization, Lab Three Handout, *United States Air Force Academy, Department of Chemistry*, **2023**.
3) Iacono ST.; Jennings AR.; Recent Studies on Fluorinated Silica Nanometer-Sized Particles. *Nanomaterials* (Basel). **2019** May 2;9(5):684.
4) Pavlo Sivolapov.; Oleksiy Myronyuk.; Denys Baklan.; Synthesis of Stober silica nanoparticles in solvent environments with different Hansen solubility parameters. *Inorganic Chemistry Communications*, **2022**. Volume 143, 109769, ISSN 1387-7003. https://doi.org/10.1016/j.inoche.2022.109769.

5) Tahereh Gholami.; Masoud Salavati-Niasari.; Mehdi Bazarganipour.; Elham Noori.; Synthesis and characterization of spherical silica nanoparticles by modified Stöber process assisted by organic ligand. *Superlattices and Microstructures*, **2013**. Volume 61, Pages 33-41, ISSN 0749-6036. https://doi.org/10.1016/j.spmi.2013.06.004.

6) Vanitha Selvarajan.; Sybil Obuobi.; Pui Lai Rachel Ee.; Silica Nanoparticles – A Versatile Tool for the Treatment of Bacterial Infections. *Frontiers in Chemistry*, **2020**.

7) Barbare Korzeniowska.; Robert Nooney.; Dorata Wencel.; Colette McDonagh.; Silica nanoparticles for cell imaging and intracellular sensing. *Nanotechnology*, **2013**. 24 (44):442002.

IOP Publishing Nanotechnology
Nanotechnology 32 (2021) 205603 (7pp) https://doi.org/10.1088/1361-6528/abe4fd

Monolayer of silica nanospheres assembled onto ITO-coated glass substrates by spin-coating

T A Faraco[1,5], N A Yoshioka[1], R M Sábio[6], H da S Barud[2], I O Maciel[1], W G Quirino[1], B Fragneaud[1], A M de Aguiar[3], S J L Ribeiro[4], M Cremona[5] and C Legnani[1,4]

[1] Grupo de Nanociência e Nanotecnologia (NANO), Departamento de Física, Universidade Federal de Juiz de Fora (UFJF), Juiz de Fora, MG, 36036-330, Brasil
[2] Laboratório de Biopolímeros e Biomateriais (BIOPOLMAT), Departamento de Biotecnologia, Universidade de Araraquara (UNIARA), Araraquara, SP, 14801-340, Brasil
[3] Departamento de Física, Universidade Federal de Juiz de Fora (UFJF), Juiz de Fora, MG, 36036-330, Brasil
[4] Institudo de Química, Universidade Estatual Paulista Júlio de Mesquita Filho (UNESP), Araraquara, SP, 14801-970, Brasil
[5] Laboratório de Optoeletrônica Molecular (LOEM), Departamento de Física, Pontifícia Universidade Católica do Rio de Janeiro (PUC-Rio), Rio de Janeiro, RJ, 22453-970, Brasil
[6] Faculdade de Ciências Farmacêuticas, Universidade Estatual Paulista Júlio de Mesquita Filho (UNESP), Araraquara, SP, 14800-903, Brasil

E-mail: thalesfaraco@gmail.com

Received 2 November 2020, revised 25 January 2021
Accepted for publication 10 February 2021
Published 26 February 2021

Abstract

In this work, we synthesized colloidal silica nanospheres with an average size of 400 nm through the modified Stöber method and successfully fabricated an ordered close-packed silica nanosphere monolayer onto ITO-coated glass substrates using a three-step spin-coating method. ITO films showed resistivity comparable to that of commercial ITO and the silica nanosphere monolayer-coated ITO/glass substrate exhibited good optical transmittance in the visible (550 nm) and near-infrared (900 nm) regions of 62% and 82%, respectively. The results suggest that this monolayer can be used in optoelectronic devices to enhance efficiency in photovoltaic cells.

Keywords: silica nanospheres monolayer, ITO substrates, SiO_2 nanospheres

(Some figures may appear in colour only in the online journal)

1. Introduction

Nanotechnology, i.e. the ability to produce and control nanostructures, is one of the most promising fields nowadays, due to its wide applicability in almost all types of industrial sectors. Nanomaterials [1] can be of inorganic or organic nature [2] and show several morphologies [3], such as nanotubes [4], nanorods [5] and spherical nanoparticles [6].

Considering the importance of surface arrangement in nanostructured materials, ordered close-packed nanoparticle monolayers assembled onto different substrates have been widely investigated [7–9]. Currently, several methods have been employed to manufacture nanosphere monolayers, although many of them can involve very expensive processes, such as nanolithography [10]. Other possible low-cost methods are spray-coating [11], dip-coating [12] and wet-coating [13] techniques. Considering this scenario, spin-coating [14] stands out as an efficient, simple, and cheap method widely used for the deposition of thin coatings onto flat substrates. In addition, it allows an efficient deposition of nanospheres without the need of lithography and with fast processability on a large scale.

 © 2021 IOP Publishing Ltd Printed in the UK

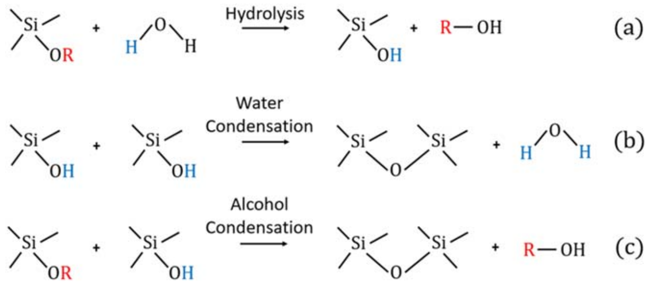

Figure 1. Hydrolysis (a) and condensation (b), (c) reactions occurring in sol–gel process, where R is alkyl.

Silica nanospheres have stood out in recent decades for nanotechnological applications, such as in electronic [15–17] and biomedical [18–21] areas, due to their chemical and physical stabilities, good biocompatibility and easy functionalization combined with a simple and low-priced synthetic route. Furthermore, preparation techniques provide easy tunability of particles size, porosity and shape [22, 23]. There are recent studies that show that the use of silica nanospheres in photovoltaic devices provides a larger light trapping, leading to an enhancement in the short-circuit current density and resulting in a performance improvement of these devices. In these works [24–29], increases in the short-circuit current density between 11.5% and 42.5% have been reported.

Tin-doped indium oxide (ITO) [30] thin film onto glass substrates possess excellent electrical conductivity and high optical transparency in the visible and near infrared regions. Because of these important characteristics, ITO-coated glass has been widely used as a conducting substrate in the manufacturing of various devices, such as organic light-emitting diodes—OLEDs [31, 32], organic photovoltaics—OPVs [33, 34] and electrochemical biosensors [35, 36].

In this work we fabricated colloidal silica nanospheres to produce an ordered close-packed silica nanosphere monolayer onto ITO-coated glass substrates by spin-coating method, aiming to manufacture a cheap platform for optoelectronic devices with increased performance.

2. Experimental details

2.1. Synthesis of colloidal silica nanospheres

The process developed by Werner Stöber [37, 38] in 1968 is one of the most relevant routes for preparation of colloidal silica nanospheres. This method consists of the hydrolysis and condensation of a silane alkoxide precursor, as shown in figure 1. The hydrolysis process forms silanol groups (figure 1(a)), followed by a condensation process between silanol groups (figure 1(b)) or between silanol and ethoxy groups (figure 1(c)), creating siloxane bridges (Si–O–Si), and the entire silica nanosphere network [39, 40].

Colloidal SiO_2 nanospheres were prepared through a sol–gel process based on the modified Stöber method [41]. Tetraethylorthosilicate ($SiC_8H_{20}O_4$, labeled TEOS) was used as inorganic precursor in an isopropyl alcoholic medium (C_3H_8O), using ammonia hydroxide (NH_4OH) as the base-catalyst. Thus, 7.2 ml of C_3H_8O (>99.7% Sigma Aldrich), 3.0 ml of deionized water (mili-Q), 1.2 ml of NH_4OH (>99.9% Sigma-Aldrich) and 0.7 ml of TEOS (>99.9% Sigma Aldrich) were added, in this order, into a 100 ml round-bottom flask. The reaction was kept in a glycerin bath (30 °C) under magnetic stirring (500 rpm) for 90 min. The colloidal suspension was centrifuged (10 000 rpm, 3 min) and re-dispersed in ethanol (95% Sigma-Aldrich) 5 times. This washing process is important to remove unreacted or residual compounds.

2.2. Preparation of ITO-coated glass substrates

Prior to the deposition of ITO thin films, glass substrates (1.25×1.25 cm^2) were submerged in a beaker containing a mixture of H_2SO_4 and H_2O_2 (3:1, v/v) at 70 °C for 10 min in order to clean their surfaces. Later, they were thoroughly washed with deionized water. Then the substrates were ultrasonicated in isopropyl alcohol for 10 min and finally dried with nitrogen gas jets.

Subsequently, ITO films (200 nm) were deposited through the rf-magnetron sputtering method (13.56 MHz) in argon gas plasma with 8 Pa working pressure and 300 sccm flow at 140 W rf-power for 25 min. The films were deposited without subsequent heat treatment. The In_2O_3: SnO_2 (90:10 wt%) targets were purchased from the Lesker company (99.99% purity) and the deposition was carried out in a MB-ProVap-5 system [42].

2.3. Preparation of silica nanosphere monolayer

The colloid containing the SiO_2 nanospheres was used to prepare the nanosphere monolayer onto ITO-coated glass substrates by using a three-step spin-coating process. We used 100 μm (50 mg ml^{-1}) of the nanosphere suspension and dropped it in the center of the ITO/glass substrates fixed on a spin-coater at atmospheric pressure and room temperature (~21 °C).

It is well known that the regulation of the spinning time, spinning speed, acceleration time between the steps, concentration, quantity and colloidal nanoparticle size are some of the most important parameters for obtaining an ordered close-packed nanosphere monolayer [43].

Figure 2 shows a schematic illustration of the three-step spin-coating method to produce ordered close-packing silica nanosphere monolayer onto ITO-coated glass substrates. In the first step, a low speed of 300 rpm s^{-1} for 10 s was used to achieve a good colloidal SiO_2 suspension distribution over the entire substrate. The second step was responsible for the nanosphere organization by using a speed of 1000 rpm s^{-1} for 240 s. The third and final step was performed using a speed of 2000 rpm s^{-1} for 20 s for the nanosphere packaging process. The nanosphere monolayers were produced using a spin-coater from Laurell, 650-23NPP.

In the first step process of deposition, centrifugal and viscosity forces are the dominant factors, which causes solution flow towards the glass substrate edge. In the second

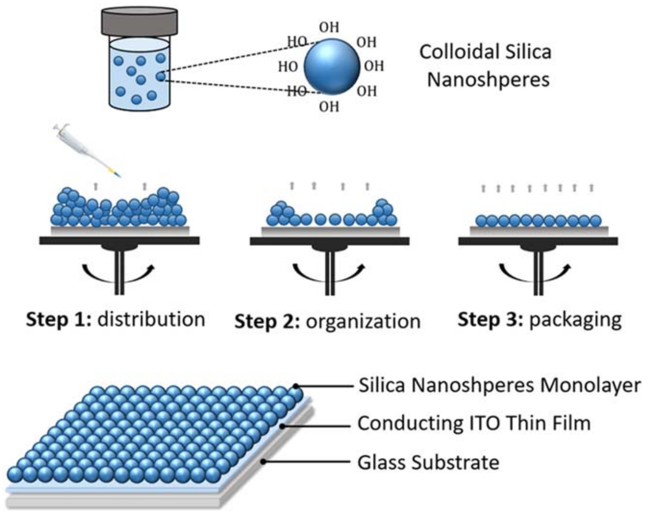

Figure 2. Schematic illustration of three-step spin-coating method to obtain an ordered close-packed silica nanosphere monolayer onto ITO-coated glass substrates.

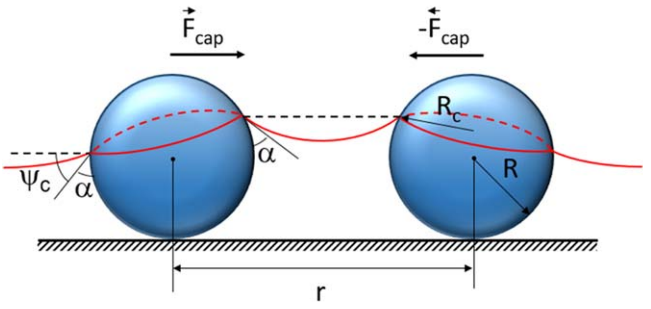

Figure 3. Capillary force in two nanospheres partially immersed in a fluid layer on a substrate.

stage, centrifugal and capillary forces start to play the main role. In the third step, the dominancy of the immersion capillary force organizing and packaging the nanospheres side by side can be observed. In this stage, the solvent evaporation also prevails [44, 45].

The capillary force intensity (F_{cap}) is essential for good ordered close-packing in the third step. This force is originated from the liquid meniscus deformation, giving rise to attraction between nanoparticles separated by a distance r, as shown in figure 3.

Thus, the module of attractive capillary force can be expressed as [46, 47]

$$F_{\text{cap}} = 2\pi\sigma R_c(\text{sen}^2\Psi_c)(1/r), \tag{1}$$

so that

$$\alpha + \Psi_c = \text{sen}^{-1}(R_c/R), \tag{2}$$

where σ is the liquid surface tension, R is the nanosphere radius, R_c is the radius of the three-phase contact line at the particle surface, α is the contact angle, ψ_c the mean meniscus (liquid capillary neck between the particle and the surface) slope angle at the contact line and r is the distance between the nanosphere centers (inter-nanosphere distances).

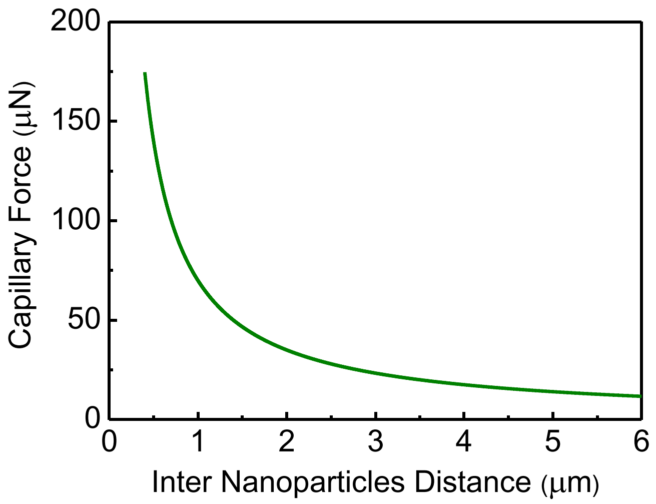

Figure 4. Capillary force plotted as a function of distance between the nanosphere centers (average particle diameter of 400 nm).

Figure 4 shows a graphic of capillary force versus inter-nanosphere distance, where the calculation was performed considering $R_c = 188$ nm (radius of 2/3 nanosphere spot), $\sigma = 4.5$ mN m^{-1} for ethanol/water mixture [47] and $\alpha = 40°$.

It is worth noting that capillary force becomes very relevant when the nanospheres are too close to each other. In this situation, the curve grows faster, reaching the highest F_{cap} value when r tends to 400 nm.

2.4. Characterization methods

Nanosphere size, zeta potential and polydispersion index of colloidal silica suspension were measured with a dynamic light scattering system (Malvern, Zetasizer Nano ZS). The thermal nanosphere behavior was studied with a thermo-gravimetric and differential thermal analyzer (Netzsch, STA 449 F3 Jupiter). ITO film thickness and roughness were analyzed with a profilometer (KLA, Tencor D-100). The structure and morphology of the silica monolayer onto ITO/glass substrate were observed through an x-ray diffractometer (Bruker, D8 Advance) and scanning electron microscope (FEI, Quanta 250). The optical properties of the SiO_2 nanospheres, ITO film and nanosphere monolayer were characterized using a spectrophotometer (Shimadzu, UV-1800). The electrical characteristics of ITO films were investigated with a Hall Effect measurement system (Ecopia, HMS-3000).

3. Results and discussion

Silica nanospheres have a spherical shape with an average diameter of 400 nm, with 10% of standard deviations. The polydispersity index was low (<0.2), indicating that the sample is homogeneous. In addition, the zeta potential analysis showed that the nanosphere surface is negatively charged, with −45.2 mV. This high module of zeta potential value (>25 mV [48]) indicates a good physicochemical stability, since large repulsive forces tend to prevent aggregation.

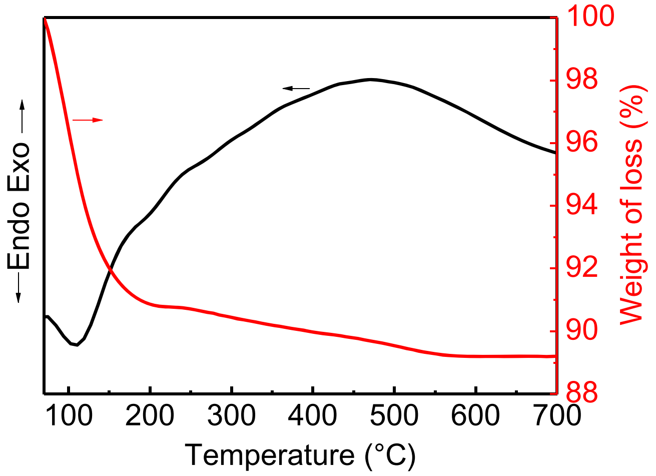

Figure 5. Thermogravimetry (TG) and differential thermal analysis (DTA) curves from SiO_2 nanospheres.

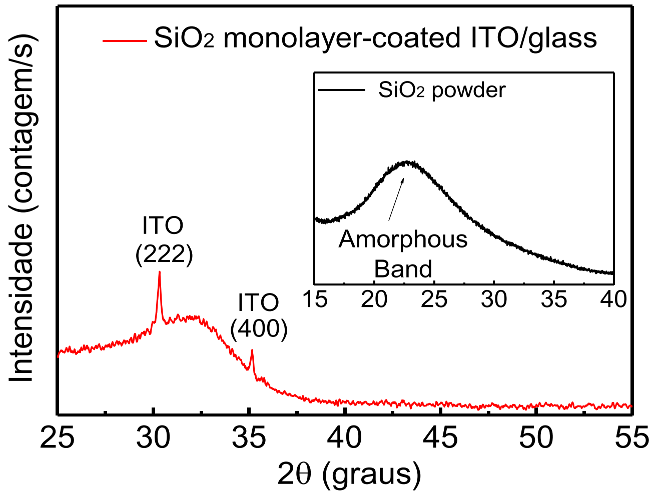

Figure 6. X-ray diffraction pattern (XRD) from silica nanosphere monolayer-coated ITO/glass substrate. Inset: silica nanosphere powder.

Table 1. Electrical properties of ITO-coated glass and ITO commercial substrates.

	Conducting substrates	
Electrical properties	ITO/glass[a]	Comm. ITO/glass[b]
N (10^{21} cm^{-3})	−0.88 ± 0.02	−1.38 ± 0.02
μ (cm^2 V^{-1} s^{-1})	10.1 ± 0.4	38.4 ± 0.4
Rs (Ω/□)	32.8 ± 0.1	11.9 ± 0.1
ρ (10^{-4} Ω cm)	7.23 ± 0.01	1.19 ± 0.01

Note. N: carrier concentration; μ: carrier mobility; R_s: sheet-resistance; ρ: electrical resistivity.
[a] 200 nm ITO film.
[b] 100 nm commercial ITO film.

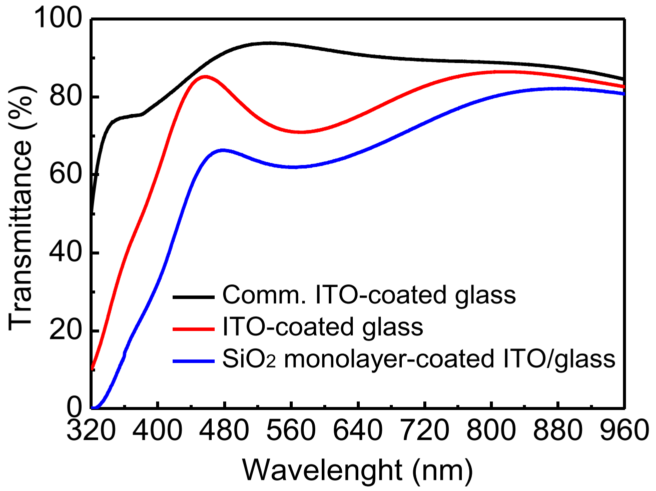

Figure 7. Optical transmittance spectrum of the ITO-coated substrate (red line), commercial ITO-coated glass substrate (black line) and silica nanosphere monolayer-coated ITO/glass substrate (blue line).

The colloidal SiO_2 suspension was dried before thermal analysis. Figure 5 exhibits the thermogravimetry (TG) and differential thermal analysis (DTA) curves for SiO_2 nanospheres.

The endothermic peak at 110 °C and the mass loss are attributed to the removal of the remaining water and ethanol present in nanoparticles powder. The mass loss at higher temperatures was attributed to the dihydroxylation of silanol groups and organic amines decomposition [49, 50]. The total mass losses, up to 700 °C, was 11%.

After analyzing the optical and thermal properties of the SiO_2 nanospheres, the electrical properties of ITO-coated glass substrate were evaluated and compared to a commercial one, as displayed in table 1.

The roughness values obtained via mean square root of the ITO-coated glass and commercial ITO-coated glass substrates were 3.6 and 2.5 nm, respectively. The roughness values were close to the commercial ITO, which is an important feature, since the roughness of the substrate surface directly interferes with the production of an ordered close-packing monolayer [51]. In addition, our ITO film showed good electrical property with an electrical resistivity of the same order (10^{-4} Ω cm^{-1}) than the commercial ITO substrates.

Figure 6 shows an x-ray diffraction pattern (XRD) of the silica nanosphere monolayer-coated ITO/glass substrates to examine the materials chemical composition and crystallinity degree. The inset exhibits XRD pattern from silica nanosphere powder, indicating they are amorphous [52].

Additionally, the diffraction peaks at 30.4° and 35.2° are attributed to the (222) and (400) crystallographic planes of the ITO cubic bixbyite polycrystalline structure [53, 54]. The XRD peak (222) is the dominant one, indicating a preferential orientation along the [111] direction, with width at half maximum (FWHM) of about 0.21°.

In order to check the substrate transparency, optical transmittance measurements were carried out. The transmission spectra for ITO-coated and commercial ITO substrates, as well as the silica nanosphere monolayer-coated ITO/glass substrate, are shown in figure 7.

The SiO_2 monolayer-coated ITO/glass substrate showed transmittance at visible (550 nm) and near-infrared (900 nm)

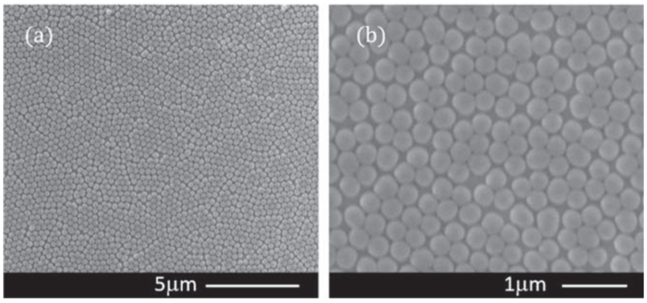

Figure 8. SEM images of silica nanosphere monolayer onto ITO/glass substrate with (a) low-magnification and (b) high-magnification.

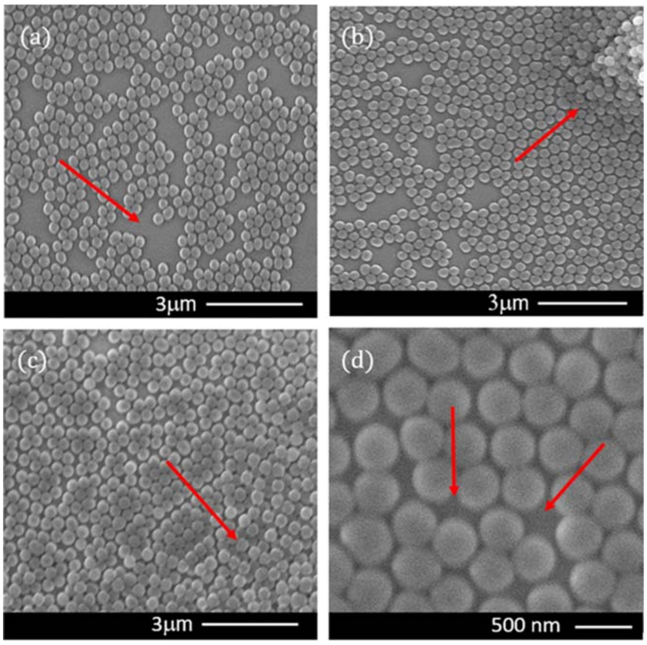

Figure 9. SEM images showing some defects found in the silica monolayer, such as: (a) absence of coverage, (b) presence of clusters, (c) formation of multiple layers and (d) line and point defects.

regions of 62% and 82%, respectively. The nanosphere monolayer reduced the transmittance of ITO glass substrate by only 10% for the visible region and 3% for the infrared region. The high transmittance in the infrared region indicates that this type of substrate can be satisfactorily used in infrared photovoltaic cells [55].

Figure 8 shows scanning electron microscopy (SEM) images of silica nanosphere monolayer onto ITO/glass substrate with two different magnitudes.

The SiO2 nanospheres, deposited onto ITO-coated glass substrates, form a homogeneous monolayer, with large surface coverage and an inherent 2D periodicity constituted by a hexagonal ordered close-packed structure consisting of seven nanospheres: six nanospheres at the vertices of the hexagon and one located at the center (see figure 8(b)).

Even though the overall coverage of the nanospheres is quite uniform, in some regions one can find some packing defects, as shown in figure 9. As examples, one can see absence of coverage (figure 9(a)), clusters of nanospheres (figure 9(b)) and formation of multiple layers (figure 9(c)). These defects can be due to undesirable impurities on the substrate that remained after the cleaning process or clusters present in silica nanosphere colloid. As these defects were found mainly in the peripheral part of the substrates (probably because of the deposition process), they would not cause major problems when developing optoelectronic devices. In the central part of the substrates, we observed only few point and line defects (figure 9(d)), which can be attributed to the difference in the nanosphere sizes and the inherent roughness of the ITO film.

4. Conclusion

In this paper, motivated to manufacture an ordered close-packed silica nanosphere monolayer onto ITO substrates, we synthesized colloidal silica nanospheres with an average size of 400 nm by using the modified Stöber method. A conducting ITO thin film onto glass substrate (1.25×1.25 cm^2) was deposited through the rf-magnetron sputtering technique, using 8 Pa and 300 sccm of argon gas atmosphere, as well as a 140 W rf-power. Finally, the successfully fabrication of silica nanosphere monolayer onto ITO-coated glass substrates using a three-step spin-coating method: 300 rpm s^{-1} for 10 s, 1000 rpm s^{-1} for 240 s and 2000 rpm s^{-1} for 20 s was achieved.

Polycrystalline ITO films showed low electrical resistivity (7.23×10^{-4} Ω cm). This film showed excellent electrical resistivity, in the same order of magnitude as that of the commercial ITO. In addition, silica nanosphere monolayer-coated ITO/glass substrate exhibited good optical transparency of 62% and 82% at visible (550 nm) and near-infrared (900 nm) regions, respectively.

We also showed that the silica nanospheres are packed in hexagonal units and discussed the importance of capillary force to obtain an ordered close-packed nanosphere monolayer, as well as some defects found in the silica monolayer.

We conclude that ordered close-packed silica nanosphere monolayer onto ITO-coated glass substrates produced the spin-coating technique, which is an inexpensive, fast and efficient method, has the potential to be used in several optoelectronic devices, such as a photovoltaic cells.

Acknowledgments

The authors would like to thank the Brazilian agencies CAPES (001), CNPq (305534/2018-1, 422539/2016-3, 407822/2018-6), FINEP (0423/15), FAPESP (CEPID-13/07276-1, 2018/25512-8, 2020/04509-9), INEO-MCT, FAPERJ and TA Instruments Brasil for the received financial support.

Data availability statement

All data that support the findings of this study are included within the article (and any supplementary files).

ORCID iDs

T A Faraco https://orcid.org/0000-0003-2957-6006
N A Yoshioka https://orcid.org/0000-0002-4348-5314
R M Sábio https://orcid.org/0000-0002-3852-2184
H da S Barud https://orcid.org/0000-0001-9081-2413
I O Maciel https://orcid.org/0000-0002-1757-4102
W G Quirino https://orcid.org/0000-0001-6294-5382
B Fragneaud https://orcid.org/0000-0001-8170-6117
A M de Aguiar https://orcid.org/0000-0003-0816-2250
S J L Ribeiro https://orcid.org/0000-0003-3286-9440
M Cremona https://orcid.org/0000-0003-1306-4639
C Legnani https://orcid.org/0000-0002-5234-5487

References

[1] Kolahalam L A, Kasi Viswanath I V, Diwakar B S, Govindh B, Reddy V and Murthy Y L N 2019 Review on nanomaterials: synthesis and applications *Mater. Today Proc.* **18** 2182–90
[2] Khalid K, Tan X, Mohd Zaid H F, Tao Y, Lye Chew C, Chu D T, Lam M K, Ho Y C, Lim J W and Chin Wei L 2020 Advanced in developmental organic and inorganic nanomaterial: a review *Bioengineered* **11** 328–55
[3] Zhang D, Zhang L, Zhang W, Huo M, Yin J, Dang G, Ren Z, Zhang Q, Xie J and Mao S S 2017 Morphology-dependent electrocatalytic performance of $Fe_2(MoO_4)_3$ for electro-oxidation of methanol in alkaline medium *J. Mater.* **3** 135–43
[4] Takakura A, Beppu K, Nishihara T, Fukui A, Kozeki T, Namazu T, Miyauchi Y and Itami K 2019 Strength of carbon nanotubes depends on their chemical structures *Nat. Commun.* **10** 1–7
[5] Chen Y S, Zhao Y, Yoon S J, Gambhir S S and Emelianov S 2019 Miniature gold nanorods for photoacoustic molecular imaging in the second near-infrared optical window *Nat. Nanotechnol.* **14** 465–72
[6] Cui J, Liang Y, Yang D and Liu Y 2016 Facile fabrication of rice husk based silicon dioxide nanospheres loaded with silver nanoparticles as a rice antibacterial agent *Sci. Rep.* **6** 21423
[7] Reissner P A, Tisserant J N, Sánchez-Ferrer A, Mezzenga R and Stemmer A 2016 Solvent-mediated conductance increase of dodecanethiol-stabilized gold nanoparticle monolayers *Beilstein J. Nanotechnol.* **7** 2057–64
[8] Chandramohan A, Sibirev N V, Dubrovskii V G, Petty M C, Gallant A J and Zeze D A 2017 Model for large-Area monolayer coverage of polystyrene nanospheres by spin coating *Sci. Rep.* **7** 40888
[9] Choi J-Y, Alford T L and Honsberg C B 2014 Solvent-controlled spin-coating method for large-scale area deposition of two-dimensional silica nanosphere assembled layers *Langmuir* **30** 5732–8
[10] Moitra P, Slovick B A, Li W, Kravchencko I I, Briggs D P, Krishnamurthy S and Valentine J 2015 Large-scale all-dielectric metamaterial perfect reflectors *ACS Photonics* **2** 692–8
[11] Rukosuyev M, Esmaeilirad A, Baqar S A and Jun M B G 2017 Uniform silver nanoparticles coating using dual regime spray deposition system for superhydrophilic and antifogging applications *J. Coat. Technol. Res.* **14** 347–54
[12] Chen T, Yang H, Bai S, Zhang Y and Guo X 2020 Facile preparation of high conductive silver electrodes by dip-coating followed by quick sintering *R. Soc. Open Sci.* **7** 1–11
[13] Hu M, Chujo S, Nishikawa H, Yamaguchi Y and Okubo T 2004 Spontaneous formation of large-area monolayers of well-ordered nanoparticles via a wet-coating process *J. Nanopart. Res.* **6** 479–87
[14] Chen J, Dong P, Di D, Wang C, Wang H, Wang J and Wu X 2013 Controllable fabrication of 2D colloidal-crystal films with polystyrene nanospheres of various diameters by spin-coating *Appl. Surf. Sci.* **270** 6–15
[15] Gollu S R, Sharma R, Srinivas G, Kundu S and Gupta D 2015 Incorporation of SiO_2 dielectric nanoparticles for performance enhancement in P3HT:PCBM inverted organic solar cells *Org. Electron.* **24** 43–50
[16] Furasova A, Calabró E, Lamanna E, Tiguntseva E, Ushakova E, Ubyivovk E, Mikhailovskii V, Zakhidov A, Makarov S and Di Carlo A 2018 Resonant silicon nanoparticles for enhanced light harvesting in halide perovskite solar cells *Adv. Opt. Mater.* **6** 1800576
[17] Kang J S, Lim J, Rho W Y, Kim J, Moon D S, Jeong J, Jung D, Choi J W, Lee J K and Sung Y E 2016 Wrinkled silica/titania nanoparticles with tunable interwrinkle distances for efficient utilization of photons in dye-sensitized solar cells *Sci. Rep.* **6** 30829
[18] Distasi C *et al* 2018 SiO_2 nanoparticles modulate the electrical activity of neuroendocrine cells without exerting genomic effects *Sci. Rep.* **8** 2760
[19] Zhao S, Zhang S, Ma J, Fan L, Yin C, Lin G and Li Q 2015 Double loaded self-decomposable SiO_2 nanoparticles for sustained drug release *Nanoscale* **7** 16389–98
[20] Mathelié-Guinlet M, Cohen-Bouhacina T, Gammoudi I, Martin A, Béven L, Delville M H and Grauby-Heywang C 2019 Silica nanoparticles-assisted electrochemical biosensor for the rapid, sensitive and specific detection of *Escherichia coli Sensors Actuators* B **292** 314–20
[21] Guzman-Ruiz M A, De La Mora M B, Torres X, Meza C, Garcia E and Chavarria A 2019 Oral silica nanoparticles lack of neurotoxic effects in a Parkinson's disease model: a possible nanocarrier? *IEEE Trans. Nanobiosci.* **18** 535–41
[22] Rahman I A and Padavettan V 2012 Synthesis of Silica nanoparticles by sol–gel: size-dependent properties, surface modification, and applications in silica-polymer nanocompositesa review *J. Nanomater.* **2012** 1–15
[23] Jeelani P G, Mulay P, Venkat R and Ramalingam C 2019 Multifaceted application of silica nanoparticles. A review *Silicon* **2019** 1–18
[24] Grandidier J, Weitekamp R A, Deceglie M G, Callahan D M, Battaglia C, Bukowsky C R, Ballif C, Grubbs R H and Atwater H A 2013 Solar cell efficiency enhancement via light trapping in printable resonant dielectric nanosphere arrays *Phys. Status Solidi* A **210** 255–60
[25] Shao P, Chen X, Guo X, Zhang W, Chang F, Liu Q, Chen Q, Li J, Li Y and He D 2017 Facile embedding of SiO_2 nanoparticles in organic solar cells for performance improvement *Org. Electron.* **50** 77–81
[26] Yang H, Ding Q, Li B Q, Jiang X and Zhang M 2018 Synergetic scattering of SiO_2 and Ag nanoparticles for light-trapping enhancement in organic bulk heterojunction *J. Nanopart. Res.* **20** 1–8
[27] Seyedpour Esmaeilzad N, Demir A K, Hajivandi J, Ciftpinar H, Turan R, Kurt H and Bek A 2020 Nanosphere concentrated photovoltaics with shape control *Adv. Opt. Mater.* **9** 2000943
[28] Wang J, Zhang H, Wang L, Yang K, Cang L, Liu X and Huang W 2020 Highly stable and efficient mesoporous and hollow silica antireflection coatings for Perovskite solar cells *ACS Appl. Energy Mater.* **3** 4484–91
[29] Luo Q, Deng X, Zhang C, Yu M, Zhou X, Wang Z, Chen X and Huang S 2018 Enhancing photovoltaic performance of perovskite solar cells with silica nanosphere antireflection coatings *Sol. Energy* **169** 128–35

[30] Tuna O, Selamet Y, Aygun G and Ozyuzer L 2010 High quality ITO thin films grown by dc and RF sputtering without oxygen *J. Phys. D: Appl. Phys.* **43**
[31] Chen Y H, Ma D G, Sun H D, Chen J S, Guo Q X, Wang Q and Zhao Y B 2016 Organic semiconductor heterojunctions: electrode-independent charge injectors for high-performance organic light-emitting diodes *Light: Sci. Appl.* **5** e16042
[32] Faraco T A *et al* 2019 Ecological biosubstrates obtained from onion pulp (Allium cepa L.) for flexible organic light-emitting diodes *ACS Appl. Mater. Interfaces* **11** 42420–8
[33] Jinno H, Fukuda K, Xu X, Park S, Suzuki Y, Koizumi M, Yokota T, Osaka I, Takimiya K and Someya T 2017 Stretchable and waterproof elastomer-coated organic photovoltaics for washable electronic textile applications *Nat. Energy* **2** 780–5
[34] Huang W, Jiang Z, Fukuda K, Jiao X, McNeill C R, Yokota T and Someya T 2020 Efficient and mechanically robust ultraflexible organic solar cells based on mixed acceptors *Joule* **4** 128–41
[35] Özcan B, Hanbaba M A and Kemal Sezgintürk M 2020 Ultra-sensitive detection of parathyroid hormone in human serum: a cheap and practical biosensing platform modified by an epoxy ended-silane agent *Int. J. Environ. Anal. Chem.* **100** 393–407
[36] Ridhuan N S, Abdul Razak K and Lockman Z 2018 Fabrication and characterization of glucose biosensors by using hydrothermally grown ZnO nanorods *Sci. Rep.* **8** 13722
[37] Stober W, Fink A and Bohn E 1968 Controlled growth of monodisperse silica spheres in the micron size range 1 *J. Colloid Interface Sci.* **26** 62–9
[38] Brito-Silva A M, Galembeck A, Gomes A S L, Jesus-Silva A J and De Araújo C B 2010 Random laser action in dye solutions containing Stöber silica nanoparticles *J. Appl. Phys.* **108** 033508
[39] Ismail W N W 2016 Sol–gel technology for innovative fabric finishing—a review *J. Sol-Gel Sci. Technol.* **78** 698–707
[40] Mestanza S N M, Ribeiro A O, Ribeiro C S de S, Giunta G and Ribera A 2017 Study of the influence of dynamics variables on the growth of silica nanoparticles *Inorg. Nano-Met. Chem.* **47** 824–9
[41] Bueno L A, Bertholdo R, Barros Filho D A, Messaddeq Y and Ribeiro S J L 2002 Rare earth doped synthetic opals and inverse opals *Sol-Gel Opt.* VI **4804** 121–9
[42] MB-ProVap-5 Mbraun 2021 (https://www.mbraun.com/us/)
[43] Ogi T, Modesto-Lopez L B, Iskandar F and Okuyama K 2007 Fabrication of a large area monolayer of silica particles on a sapphire substrate by a spin coating method *Colloids Surf.* A **297** 71–8
[44] Shinde S S, Park S and Shin J 2015 Spin synthesis of monolayer of SiO_2 thin films *J. Semicond.* **36** 043002
[45] Wang Q D, Ye L, Wang L, Li P Y, Cao Y and Li Y 2016 Rapid nanopatterning technique based on monolayer silica nanosphere close-packing by spin coating *Sci. China Technol. Sci.* **59** 1573–80
[46] Velev O D, Denkov N D, Kralchevsky P A, Ivanov I B, Yoshimura H and Nagayama K 1992 Mechanism of formation of two-dimensional crystals from latex particles on substrates *Langmuir* **8** 3183–90
[47] Khanna S, Utsav, Marathey P, Chaliyawala H, Rajaram N, Roy D, Banerjee R and Mukhopadhyay I 2018 Fabrication of long-ranged close-packed monolayer of silica nanospheres by spin coating *Colloids Surf.* A **553** 520–7
[48] Vedula V B, Chopra M, Joseph E and Mazumder S 2016 Preparation and characterization of nanoparticles of carboxymethyl cellulose acetate butyrate containing acyclovir *Appl. Nanosci.* **6** 197–208
[49] Nandy S, Kundu D and Naskar M K 2014 Synthesis of mesoporous Stöber silica nanoparticles: the effect of secondary and tertiary alkanolamines *J. Sol-Gel Sci. Technol.* **72** 49–55
[50] Raileanu M, Todan L, Crisan M, Braileanu A, Rusu A, Bradu C, Carpov A and Zaharescu M 2010 Sol–gel materials with pesticide delivery properties *J. Environ. Prot.* **1** 302–13
[51] Ţălu Ş, Morozov I A and Yadav R P 2019 Multifractal analysis of sputtered indium tin oxide thin film surfaces *Appl. Surf. Sci.* **484** 892–8
[52] Morga M, Adamczyk Z and Kosior D 2017 Silica nanoparticle monolayers on a macroion modified surface: formation mechanism and stability *Phys. Chem. Chem. Phys.* **19** 22721–32
[53] Gulen M, Yildirim G, Bal S, Varilci A, Belenli I and Oz M 2013 Role of annealing temperature on microstructural and electro-optical properties of ITO films produced by sputtering *J. Mater. Sci., Mater. Electron.* **24** 467–74
[54] Malathy V, Sivaranjani S, Vidhya V S, Prince J J, Balasubramanian T, Sanjeeviraja C and Jayachandran M 2009 Amorphous to crystalline transition and optoelectronic properties of nanocrystalline indium tin oxide (ITO) films sputtered with high rf power at room temperature *J. Non-Cryst. Solids* **355** 1508–16
[55] Dai J, Jiang X, Wang H and Yan D 2007 Organic photovoltaic cells with near infrared absorption spectrum *Appl. Phys. Lett.* **91** 253503
